# Cervical cancer burden and attributable risk factors across different age and regions from 1990 to 2021 and future burden prediction: results from the global burden of disease study 2021

**DOI:** 10.3389/fonc.2025.1541452

**Published:** 2025-02-07

**Authors:** Lu-yao Cheng, Ji-qi Zhao, Ting-ting Zou, Zhong-hua Xu, Yin Lv

**Affiliations:** ^1^ Department of Oncology Radiotherapy, The First Affiliated Hospital of Anhui Medical University, Hefei, Anhui, China; ^2^ The Center for Scientific Research, The First Affiliated Hospital of Anhui Medical University, Hefei, Anhui, China

**Keywords:** cervical cancer, global burden of disease, disability-adjusted life-years, incidence, mortality

## Abstract

**Background:**

Cervical cancer (CC) is a global public health problem. We aimed to evaluate the global and regional CC burden between 1990 and 2021, identify the attributable risk factors, and project its burden up to 2035.

**Methods:**

Data were extracted from the Global Burden of Disease Study 2021, and the CC incidence, mortality, age-standardized incidence rate (ASIR), age-standardized death rate (ASDR), age-standardized disability-adjusted life years (DALYs), and attributable risk factors from 1990 to 2021 were analyzed. The impacts of geographical variations, different age groups, and the socio-demographic index (SDI) on CC morbidity and mortality measurements were assessed. The attributable risk factors to CC death and DALY were evaluated, and the incidence, mortality, and DALYs to 2035 were projected.

**Results:**

Globally, the number of CC cases has increased from 409,548.49 cases in 1990 to 667,426.40 cases in 2021. However, the ASIR decreased from 18.11 to 15.32 per 100,000, with the greatest ASIR decrease in high SDI regions (estimated annual percentage change: -1.41). Between 1990 and 2021, the global ASDR decreased from 9.68 to 6.62 per 100,000, and the rate of age-standardized DALYs decreased from 330.11 to 226.28 per 100,000. However, these improvements were not consistent across different SDI regions. The CC incidence was the highest in the 55-59 age group, globally. The risk factors, which included unsafe sex and smoke, significantly varied by region. The global ASIR exhibited a downward trend from 2021 to 2035.

**Conclusion:**

From 1990 to 2021, although the overall trend in incidence, mortality, and DALYs of CC exhibited a global and regional downward trend, there were significant disparities among areas with different socioeconomic development. More efficient targeted prevention and management strategies, easy access to health care in less developed regions, and risk factor modifications should be promoted, in order to reduce the global burden of CC.

## Introduction

Cervical cancer (CC) is one of the most prevalent gynecologic malignancies (1). Despite the global efforts to eliminate CC, this remains as the fourth leading cause of cancer death in women, with nearly 660,000 new cases and 350,000 deaths reported in 2022 (2). Almost all CC cases were caused by infection from human papillomaviruses (HPVs) (3). HPV vaccine (primary prevention) and CC screening (secondary prevention) have been proven to effectively decrease the CC incidence, and improve its early detection and treatment, thereby changing the epidemiological pattern of CC (4). However, these preventive strategies are not uniform across different regions, which might be due to socioeconomic and geographical variations, as well as different cultural backgrounds and local healthcare infrastructure (5). The difference in these preventive strategies might explain the significant disparities in CC morbidity and mortality across different countries (6). In addition, other variables, such as age, race, smoking, sexual behavior, and nutritional status, can contribute to CC risk, and cause distinct regional CC characteristics (7–9). Understanding the regional characteristics of CC burden, and identifying CC contributing risk factors can facilitate the development of effective prevention measures, and appropriate allocation of medical resources.

The Global Burden of Disease (GBD) study 2021 provides a large-scale systematic framework that quantifies the common disease status and risk factors in different regions, spanning 204 countries and territories from 1990 to 2021 (10). The GBD study 2021 aims to quantify health issues from multiple diseases and risk factors worldwide, and provide insights into the overall impact of different health challenges, in order help researchers, policymakers, and organizations in prioritizing and implementing interventions. Several recent studies have analyzed the GBD study 2021 to report the influence of age, the socio-demographic index (SDI), and geographical location on breast cancer, gastrointestinal cancers, and respiratory system malignancies (11–13). However, a similar analysis on CC has never been reported, despite CC being a significant public health issue (14).

The present study analyzed the GBD study 2021, and characterized the influence of geographic location, SDI, and age on global and regional trends in CC morbidity, mortality, and disability-adjusted life-years (DALYs). In addition, the projections of CC burden in 2035 from the GBD data were further assessed, with the objective of providing up-to-date epidemiologic data and analysis, in order to facilitate CC prevention and management.

## Materials and methods

### Data source and disease definition

The GBD study 2021 was the primary data source for the present research. The materials and methods for the data collection and analysis have been previously described (15, 16). The study protocol was approved, and the informed consent was waived by the University of Washington Institutional Review Board. The present study was reported according to the Strengthening the Reporting of Observational Studies in Epidemiology guidelines (17).

The GBD study 2021 data was accessed through the Institute for Health Metrics and Evaluation website (https://vizhub.healthdata.org/gbd-results/). The annual statistics on CC incidence, mortality, DALYs, relevant age-standardized rates (ASRs), and 95% uncertainty intervals (UIs) were extracted. DALY due to CC was defined as the sum of the number of years of life lived with disability (DALYs) and years of life lost.

### Socio-demographic index

The SDI is a quantitative measurement to evaluate the local socioeconomic development level. This is based on three key variables: total education attainment, fertility rate, and income per capita. The score ranges between 0 and 1, with a higher score corresponding to advanced socioeconomic development (18, 19). The SDI can indicate the disease burden and mortality rate. The present study used five categories for the SDI to assess the correlation between socioeconomic development and CC: high, medium-high, medium, low-medium, and low index.

### Risk factors

The present study analyzed the relationship between risk factors and CC, and determined whether these risk factors were involved in CC-related DALYs and mortality. Then, this relationship was further stratified by different regions to assess any geographical variations, which might determine the region-specific risk factors for targeted preventive strategies. The regional classification was provided by the GBD study 2021 (20).

### Bayesian age-period-cohort model

The Bayesian age-period-cohort (BAPC) model was applied to predict the CC incidence and death rates in 2035 (21). These were categorized into every 5-year age group, in order to provide valuable information for future CC prevention and public health initiatives.

### Statistical analysis

CC burden was quantified using age-standardized incidence rates (ASIRs), age-standardized death rates (ASDRs), and age-standardized DALY rates. In addition, the incidence, DALYs, and mortality were documented. The data were reported in estimates with 95% UIs (19). ASR was calculated using the following formula:


$$ASR=\frac{\sum_{i=1}^{A}a_{i}w_{i}}{\sum_{i=1}^{A}w_{i}}\times 100,000$$

Where: *A* refers to the number of age groups, *w_i_* refers to the number of individuals in the *i^th^* age group of the standard population, and *a_i_* refers to the age-specific rate in the *i^th^* age group.

In order to evaluate the trends over time in CC incidence and mortality, the estimated annual percentage change (EAPC) with its 95% confidence interval (CI) was calculated using a linear regression model (22):


$$\text{y}=\alpha +\beta \text{χ}+\epsilon \text{EAPC}=100{\;}^{*}\;(\text{exp}(\beta )-1)$$

An EAPC 95% CI value of 0 suggests no significant variation in CC burden over time. Otherwise, a lower limit of 95% CI of >0 would indicate a statistically significant upward trend, while an upper limit of 95% CI of <0 would indicate a statistically significant downward trend.

The smoothing splines model was employed to assess the potential correlation between CC burden and SDI in different regions and countries (23). The calculation considered the disease rates and SDI in different locations. The smooth splines model automatically selected the number, degree, and knots based on the data and span, after being fitted by the Locally Weighted Scatterplot Smoothing method. In addition, Spearman’s correlation analysis was used to evaluate the relationship between ASR and SDI, and the *r* and *p* values were reported.

Next, the BAPC model was applied to determine the multiplicative effects of age, time period, and cohort on the CC outcomes (24, 25):


$$\eta_{ij}=\mu +\alpha_{i}+\beta_{j}+\gamma_{k}$$

Where: *η_ij_* refers to the ASR; *µ* refers to the intercept; *α_i_* refers to the age effects; *β_j_* refers to the period effects, and *γ_k_* refers to the cohort effects.

The R software package (version 4.2.3) and JD_GBDR (version 2.24, Jingding Medical Technology Co., Ltd., China) were used for the statistical analyses. A *p*-value of <0.05 was considered statistically significant.

## Results

### Global and local cervical cancer incidence burden

**Figure 1:**
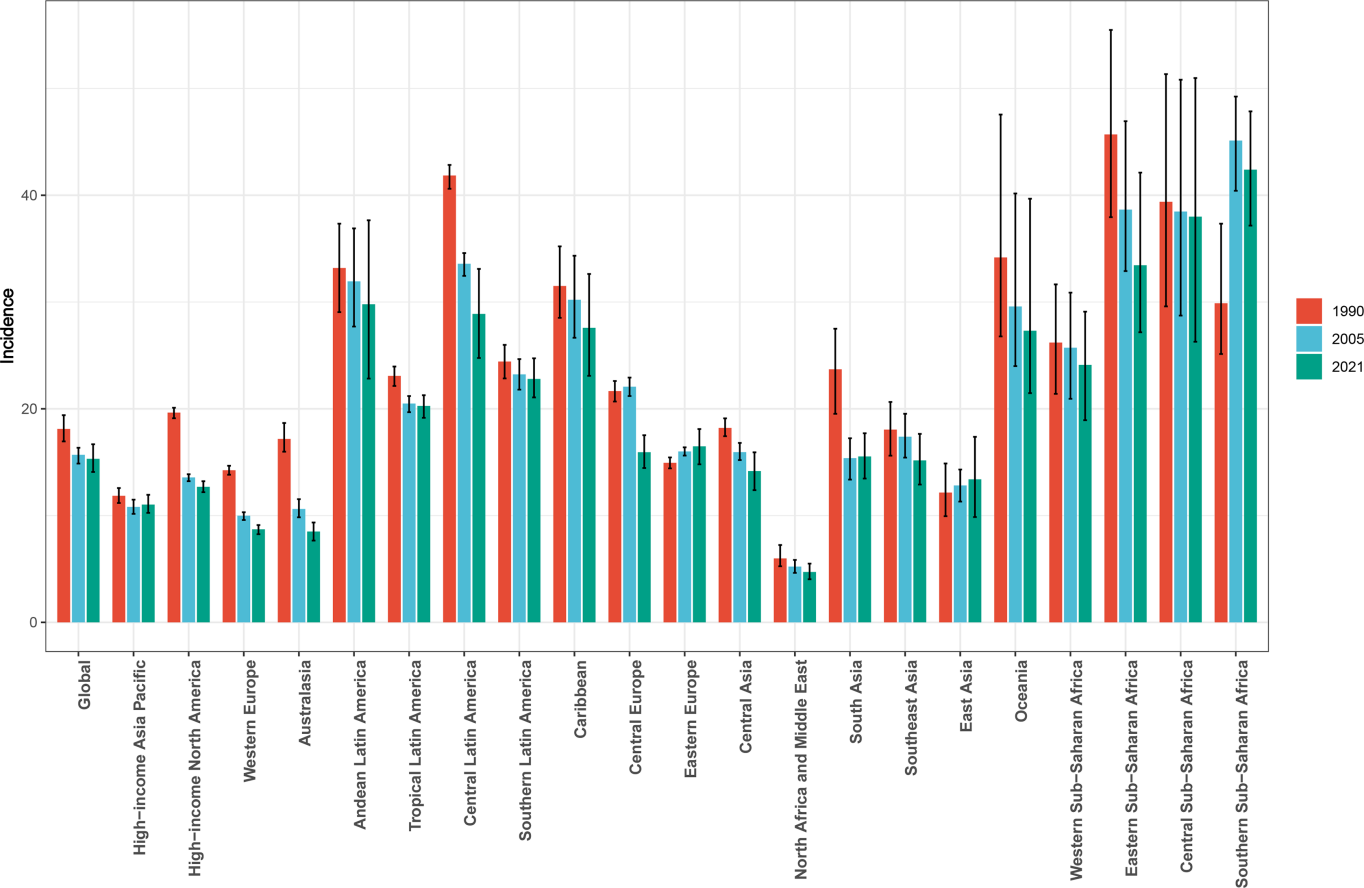
Global and local trends in the age-standardized incidence rate of cervical cancer from 1990 to 2021.

The global CC burden is presented in **Table 1** and **Figure 1**. The CC incidence significantly increased from 409,548.49 cases (95% UI: 383,207.24, 438,505.58) in 1990 to 667,426.40 cases (95% UI: 613,030.09, 726,422.07) in 2021. The global ASIR decreased from 18.11 per 100,000 people in 1990 to 15.32 per 100,000 people in 2021, with a slightly downward trend (EAPC: -0.54; 95% CI: -0.64, -0.44). In addition, the number of CC cases decreased in all five SDI regions in 2021, when compared to 1990, with the smallest number of CC cases in the high SDI region (78,008.29; 95% UI: 73,679.24, 81,040.63), and the biggest number of CC cases in the medium SDI region (228,239.87; 95% UI: 204,617.05, 254,212.15). In 2021, the ASIR was the lowest in the high SDI region (10.30 per 100,000; 95% UI: 9.91, 10.66), and the highest in the low SDI region (25.47 per 100,000; 95% UI: 21.57, 30.12). From 1990 to 2021, the ASIR exhibited a decreasing trend in most of the SDI regions, with the biggest ASIR reduction in the high SDI region (EAPC: -1.41; 95% CI: -1.57, -1.25), followed by the low SDI region (EAPC: -1.17; 95% CI: -1.29, -1.05). The only region that presented a slight increase was the medium-high SDI region (EAPC: 0.21; 95% CI: 0.11, 0.30).

**Table 1:** Global and regional incidences of cervical cancer in 1990 and 2021.

Location	1990		2021		EAPC of ASIR (95% CI)
	*n* (95% UI)	ASIR (95% UI)	*n* (95% UI)	ASIR (95% UI)	
Global	409,548.49 (383,207.24, 438,505.58)	18.11 (16.94, 19.40)	667,426.40 (613,030.09, 726,422.07)	15.32 (14.08, 16.68)	-0.54 (-0.64, -0.44)
East Asia	61,909.14 (50,407.48, 76,000.79)	12.16 (9.94, 14.88)	137,863.79 (101,144.48, 177,754.73)	13.40 (9.86, 17.38)	0.73 (0.56, 0.90)
Southeast Asia	30,364.63 (26,327.24, 34,797.64)	18.06 (15.61, 20.64)	58,017.08 (49,190.88, 67,747.07)	15.17 (12.91, 17.65)	-0.78 (-0.89, -0.67)
Oceania	668.28 (506.09, 927.96)	34.17 (26.78, 47.56)	1,372.16 (1,070.23, 2,017.14)	27.31 (21.47, 39.68)	-0.80 (-0.87, -0.74)
Central Asia	5,168.85 (4,951.55, 5,420.21)	18.21 (17.43, 19.11)	7,178.83 (6,265.57, 8,081.45)	14.17 (12.38, 15.92)	-0.45 (-0.61, -0.28)
Central Europe	16,319.82 (15,590.57, 17,021.33)	21.66 (20.68, 22.61)	14,203.02 (12,928.32, 15,545.24)	15.93 (14.45, 17.53)	-1.10 (-1.32, -0.88)
Eastern Europe	23,498.87 (22,662.27, 24,258.84)	14.94 (14.42, 15.45)	25,339.24 (22,882.94, 27,756.01)	16.49 (14.80, 18.10)	0.31 (0.19, 0.43)
High-income Asia Pacific	12,720.39 (12,005.79, 13,515.63)	11.85 (11.18, 12.58)	15,577.84 (14,044.14, 16,881.51)	11.03 (10.25, 11.95)	0.02 (-0.09, 0.13)
Australasia	2,001.17 (1,863.27, 2,175.21)	17.18 (15.98, 18.67)	1,751.57 (1,575.82, 1,924.65)	8.51 (7.66, 9.35)	-1.92 (-2.19, -1.66)
Western Europe	35,616.36 (34,385.58, 36,705.95)	14.25 (13.83, 14.66)	27,921.53 (26,003.05, 29,390.64)	8.71 (8.25, 9.11)	-1.27 (-1.49, -1.05)
Southern Latin America	6,030.06 (5,640.92, 6,407.26)	24.43 (22.84, 25.99)	9,302.11 (8,566.12, 10,068.30)	22.80 (21.07, 24.73)	-0.22 (-0.38, -0.05)
High-income North America	32,695.39 (31,769.19, 33,479.69)	19.65 (19.11, 20.10)	30,415.22 (29,096.13, 31,621.76)	12.69 (12.19, 13.22)	-1.34 (-1.56, -1.12)
Caribbean	4,635.27 (4,190.58, 5,197.10)	31.51 (28.52, 35.21)	7,381.73 (6,192.21, 8,712.12)	27.58 (23.09, 32.63)	-0.45 (-0.53, -0.37)
Andean Latin America	4,153.89 (3,640.42, 4,664.88)	33.19 (29.05, 37.34)	9,757.16 (7,475.55, 12,359.00)	29.79 (22.83, 37.66)	-0.65 (-0.82, -0.47)
Central Latin America	22,434.36 (21,858.37, 23,000.31)	41.85 (40.60, 42.84)	40,342.98 (34,553.62, 46,252.04)	28.89 (24.76, 33.10)	-1.58 (-1.77, -1.40)
Tropical Latin America	13,345.89 (12,840.53, 13,859.23)	23.08 (22.14, 23.96)	27,822.95 (26,275.18, 29,199.26)	20.27 (19.16, 21.27)	-0.85 (-1.02, -0.67)
North Africa and Middle East	6,235.20 (5,462.15, 7,543.85)	5.98 (5.25, 7.24)	12,912.91 (11,008.41, 15,181.60)	4.72 (4.04, 5.50)	-0.73 (-0.78, -0.68)
South Asia	83,506.85 (69,576.79, 96,968.91)	23.70 (19.53, 27.50)	132,481.99 (114,540.70, 151,294.70)	15.54 (13.47, 17.71)	-1.48 (-1.81, -1.14)
Central Sub-Saharan Africa	6,099.58 (4,558.31, 7,964.20)	39.39 (29.59, 51.35)	15,328.20 (10,593.80, 20,548.58)	38.00 (26.28, 50.98)	-0.17 (-0.20, -0.14)
Eastern Sub-Saharan Africa	22,644.14 (18,815.53, 27,483.05)	45.69 (37.95, 55.48)	41,370.05 (33,124.04, 52,883.49)	33.45 (27.17, 42.12)	-1.34 (-1.47, -1.22)
Southern Sub-Saharan Africa	5,528.15 (4,617.70, 6,832.39)	29.89 (25.13, 37.34)	16,246.67 (14,189.00, 18,417.61)	42.40 (37.16, 47.85)	1.89 (1.39, 2.40)
Western Sub-Saharan Africa	13,972.20 (11,424.74, 17,090.41)	26.20 (21.40, 31.65)	34,839.36 (26,668.20, 42,612.87)	24.11 (18.93, 29.10)	-0.24 (-0.28, -0.20)
High-middle SDI	73,627.86 (68,686.88, 78,719.76)	13.37 (12.47, 14.29)	120,152.64 (103,649.16, 137,728.22)	13.27 (11.44, 15.16)	0.21 (0.11, 0.30)
High SDI	87,377.11 (84,816.71, 89,248.90)	16.43 (16.04, 16.77)	78,008.29 (73,679.24, 81,040.63)	10.30 (9.91, 10.66)	-1.41 (-1.57, -1.25)
Low-middle SDI	84,749.50 (73,563.28, 97,338.25)	22.48 (19.49, 25.79)	152,719.87 (136,720.61, 169,313.95)	17.79 (15.94, 19.70)	-0.78 (-0.93, -0.63)
Low SDI	48,872.44 (41,626.59, 59,339.78)	34.34 (29.27, 41.62)	87,632.63 (73,984.19, 103,871.77)	25.47 (21.57, 30.12)	-1.17 (-1.29, -1.05)
Middle SDI	114,366.73 (105,116.99, 123,880.00)	18.09 (16.67, 19.56)	228,239.87 (204,617.05, 254,212.15)	15.94 (14.30, 17.75)	-0.47 (-0.54, -0.39)

95% CI, 95% confidence interval; 95% UI, 95% uncertainty interval; ASIR, age-standardized incidence rate; EAPC, estimated annual percentage change; SDI, social-demographic index.

Regionally, an ASIR increase from 1990 to 2021 was observed in merely three of 21 regions, including Southern Sub-Saharan Africa, Eastern Europe, and East Asia. The remaining 18 regions presented with an ASIR decrease, with Australasia having the biggest reduction (EAPC: -1.923; 95% CI: -2.188, -1.657) (**Table 1**, **Figure 1**). The highest ASIR was identified in Southern Sub-Saharan Africa (42.40 per 100,000; 95% UI: 37.16, 47.85), followed by Central Sub-Saharan Africa (38.00 per 100,000; 95% UI: 26.28, 50.98). In contrast, Australasia had a significantly lower ASIR of 8.51 per 100,000 (95% UI: 7.66, 9.35) (**Table 1**).

**Figure 2:**
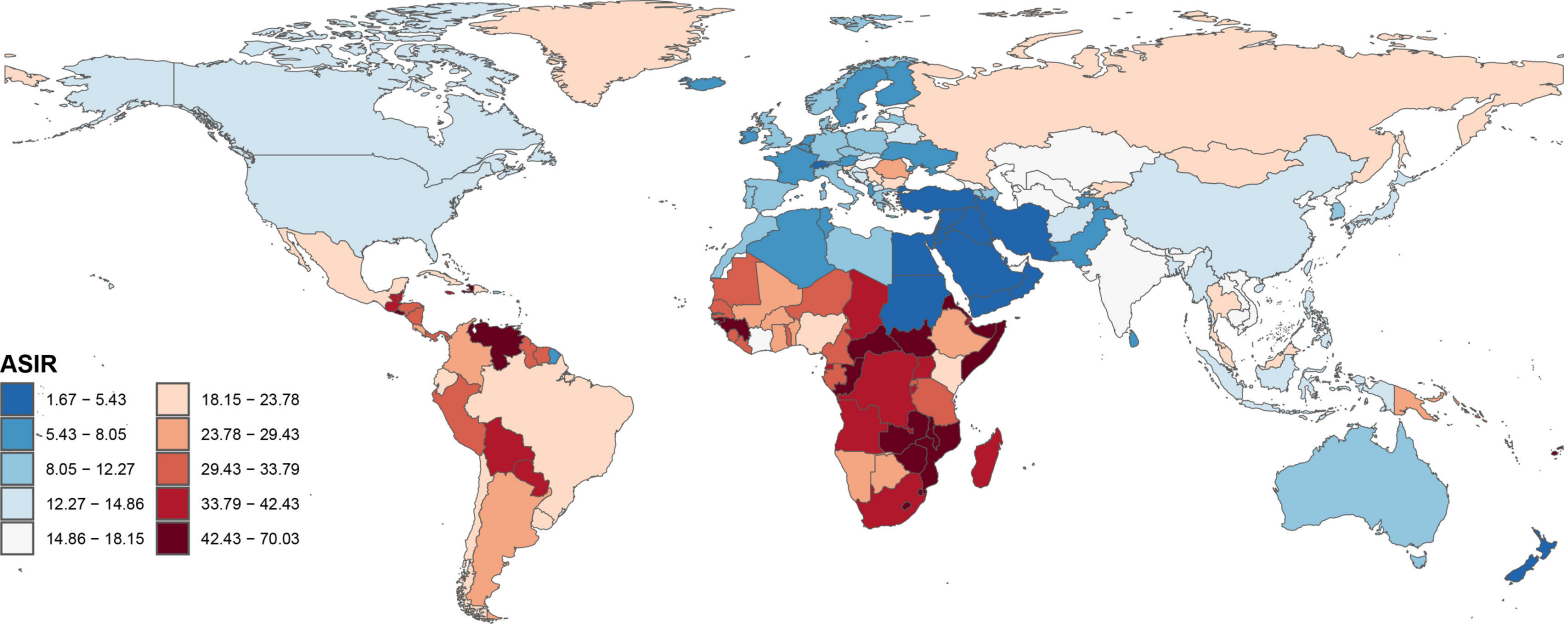
Global disease burden of cervical cancer age-standardized incidence rate for the 204 countries and territories.

**Figure 3:**
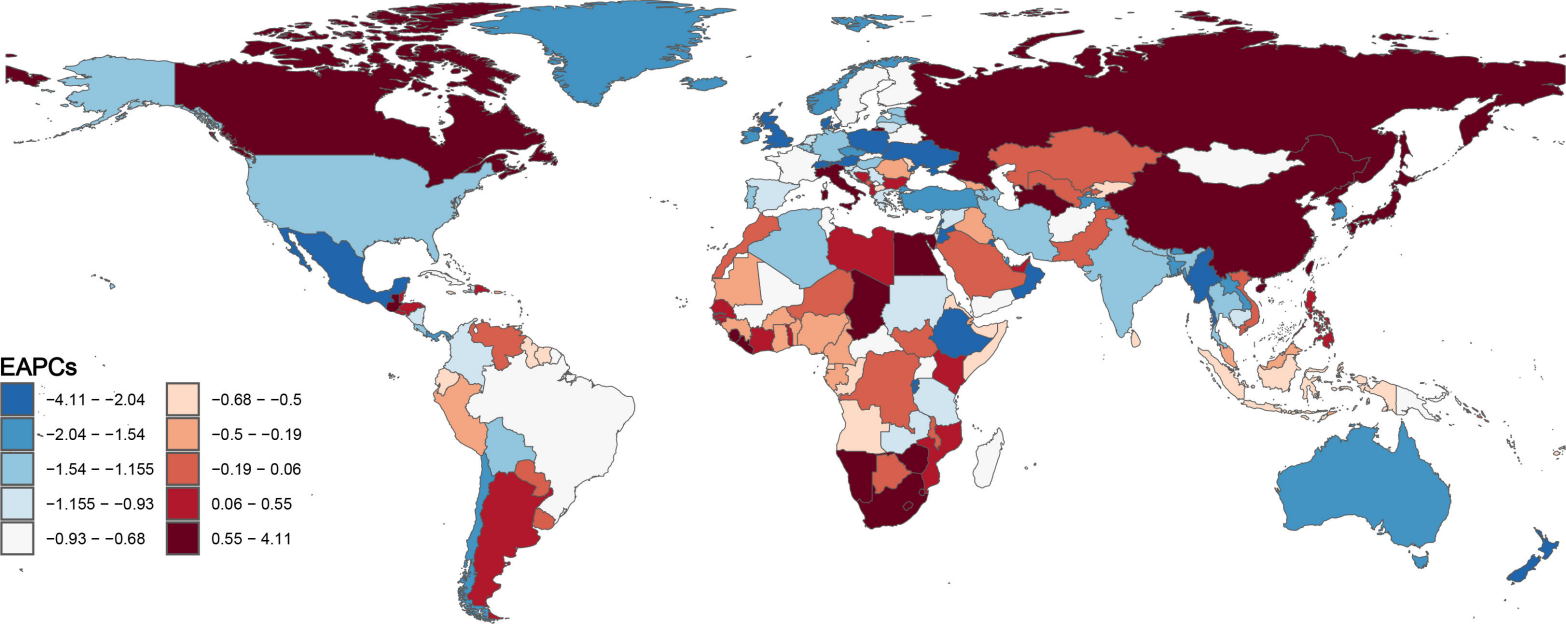
Global disease burden of cervical cancer estimated annual percentage change of age-standardized incidence rate for the 204 countries and territories from 1990 to 2021.

In terms of countries, China, Brazil, and the Russian Federation had the highest number of newly reported CC cases, while Tokelau, Niue, and San Marino had the lowest number of reported cases in 2021 (**Supplementary Table S1**). Furthermore, in 2021, Kiribati, Lesotho and Zimbabwe had the highest ASIR values, while Palestine, Kuwait and Iran (Islamic Republic of) had the lowest ASIR values (**Figure 2**). Moreover, Lesotho, Italy and Egypt had the highest increase in ASIR, while Taiwan, Maldives and Kuwait had the largest decrease in ASIR from 1990 to 2021 (**Figure 3**).

### Cervical cancer mortality and disability-adjusted life years

In 2021, the global number of CC mortality was 296,667.24 (95% UI: 272,058.62, 321,905.72) (**Supplementary Table S2**, **Supplementary Figure S1**), with an ASDR of 6.62 per 100,000 (95% UI: 6.07, 7.18). From 1990 to 2021, the ASDR decreased by 3.06 per 100,000 (**Supplementary Figures S2**, **S3**). In 2021, the global CC DALY was 9,911,653.09 (95% UI: 9,053,316.79, 10,798,305.83), with an age-standardized DALY rate of 226.28 per 100,000 (95% UI: 206.51, 246.86) (**Supplementary Figure S4**). From 1990 to 2021, the age-standardized DALY rate was reduced by 103.83 per 100,000 (**Supplementary Table S3**, **Supplementary Figures S5**, **S6**).

All five regions presented with decreased age-standardized DALY rates and ASDR over time. Compared to 1990, both age-standardized DALY rates and ASDR most significantly decreased in the high SDI region (EAPC: -2.05, 95% CI: -2.16, -1.93 for ASDR and EAPC: -1.99, 95% CI: -2.10, -1.88 for DALY). In 2021, both the highest age-standardized DALY rate (535.11, 95% UI: 454.02, 638.34) and highest ASDR (16.36, 95% UI: 13.94, 19.38) were observed in the low SDI region (**Supplementary Tables S2**, **S3**).

During the study period, the highest number of CC deaths occurred in South Asia (reaching 70,314.60, 95% UI: 61,026.36, 79,858.14, in 2021), which was followed by East Asia (52,032.08, 95% UI: 39,399.83, 66,509.90, in 2021) (**Supplementary Table S2**). Among the 21 GBD regions, Central Sub-Saharan Africa had the highest ASDR (25.10, 95% UI: 17.45, 33.97) and age-standardized DALY rate (813.59, 95% UI: 562.84, 1,104.29) in 2021 (**Supplementary Tables S2**, **S3**). The lowest ASDR and age-standardized DALY rate were observed in Australasia (ASDR: 1.85, 95% UI: 1.66, 2.02; age-standardized DALY rate: 61.68, 95% UI: 56.34, 67.50). The ASDR and age-standardized DALY rate decreased in all GBD regions, except for Southern Sub-Saharan Africa, which increased, with an ASDR of 23.90 (95% UI: 21.02, 26.67), an EAPC of ASDR of 1.71 (95% CI: 1.22, 2.19), and an age-standardized DALY rate of 1.71 (95% UI: 1.17, 2.26). Meanwhile, Australasia had the most significant reduction in CC ASDR and age-standardized DALY rates from 1990 to 2021, with an EAPC of -3.21 (95% CI: -3.44, -2.97) for ASDR and an EAPC of -3.13 (95% CI: -3.40, -2.85) for age-standardized DALY rates. Geographically, Central Europe had the highest EAPC of 6,589.55 (95% UI: 6,054.53, 7,118.26) per 100,000 people, followed by Western Europe, with 10,362.27 (95% UI: 9,191.61, 11,142.66) per 100,000 people.

Among the 204 countries in 2021, India had the highest number of CC-related mortality (60,040.82, 95% UI: 51,584.35, 69,062.11), followed by China (49,841.19, 95% UI: 36,878.07, 64,386.31) (**Supplementary Table S1**). Kiribati had the highest ASDR (45.10, 95% UI: 32.22, 58.73), while the Republic of San Marino and Saudi Arabia had the lowest ASDR (0.89, 95% UI: 0.55, 1.39; 0.89, 95% UI: 0.66, 1.17; respectively) (**Supplementary Table S1**).

### Association between age-standardized rates and the social-demographic index

**Figure 4:**
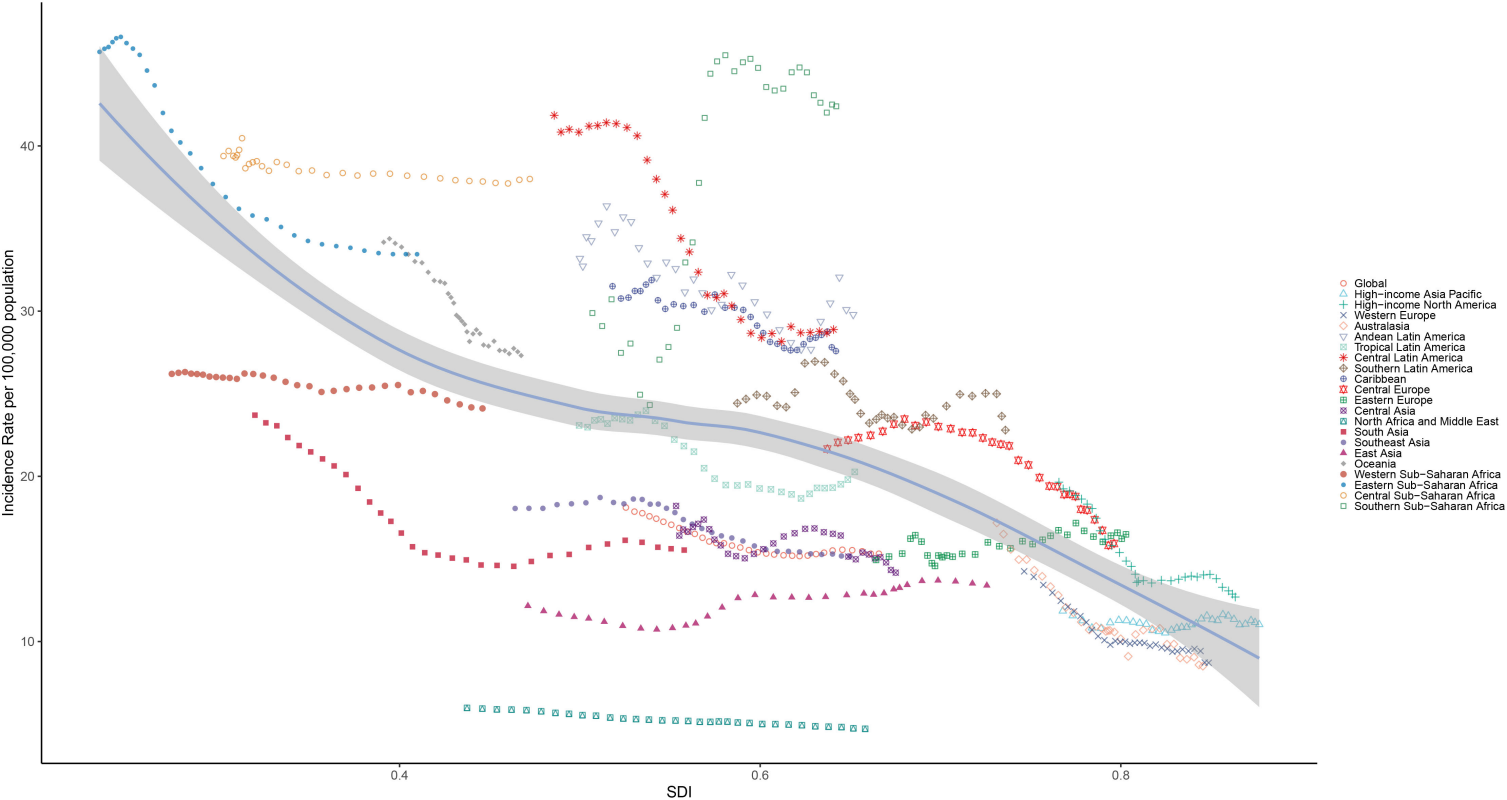
Correlation between the age-standardized incidence rate and social-demographic index in the 21 regions from 1990 to 2021.

In all 21 GBD regions, the high SCI was correlated to the low ASIR (R: -0.60, *p*<0.001) (**Figure 4**), indicating that regions with a lower SDI and less economic development were more likely to have a higher ASIR value, when compared to regions with a higher SDI and more advanced economic development. In the past 32 years, more than half of the regions (52%) had a higher-than-expected CC prevalence.

A significant negative correlation was observed between SDI, ASDRs and DALYs (R: -0.78, *p*<0.001), indicating a high CC incidence in less economically developed countries (**Supplementary Figures S7**, **S8**). Regions with a high SDI tended to have lower ASDRs and DALYs, when compared to regions with a lower SDI. Over the past 32 years, less than half of the regions (43%) had higher than expected CC death rates.

**Figure 5:**
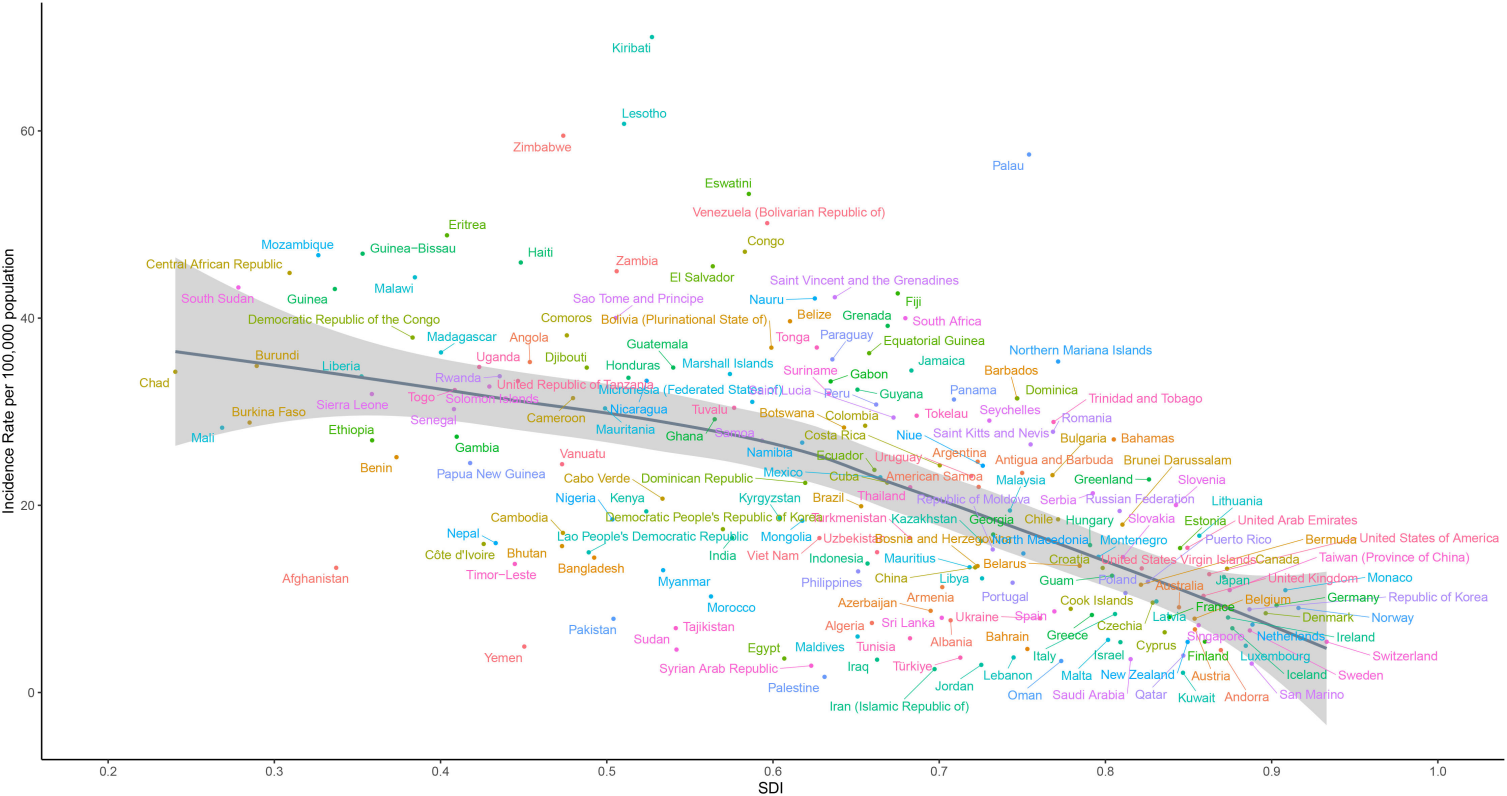
Correlation between the age-standardized incidence rate and social-demographic index in the 204 countries and territories from 1990 to 2021.

For the 204 countries and territories, SDI was negatively correlated to ASIR (R: -0.60, *p*<0.001), indicating a lower CC incidence in more economically developed areas (**Figure 5**). Countries with a higher SDI were more likely to have a lower ASIR, when compared to countries with a lower SDI. In addition, there was a negative correlation between SDI and ASDR (R: -0.74, *p*<0.001), indicating the lower incidence of CC in more economically developed countries (**Supplementary Figure S9**). Countries with a higher SDI tended to have a lower ASDR, when compared to countries with a lower SDI.

### Association between estimated annual percentage change and the social-demographic index

There was a moderate positive correlation between the EAPC of ASIR and ASIR (R: 0.4, *p*<0.001), suggesting that the ASIR increase in areas with increased ASIR (EAPC >0) further increased with the increase in ASIR, while the ASIR decrease in areas with decreased ASIR (EAPC <0) further decreased with the decrease ASIR. Furthermore, there was an overall weak negative correlation between the EAPC of ASIR and SDI (R: -0.28, *p*<0.001). The reduction in ASIR was significant with the SDI increase in areas with SDI >0.6.

**Figure 6:**
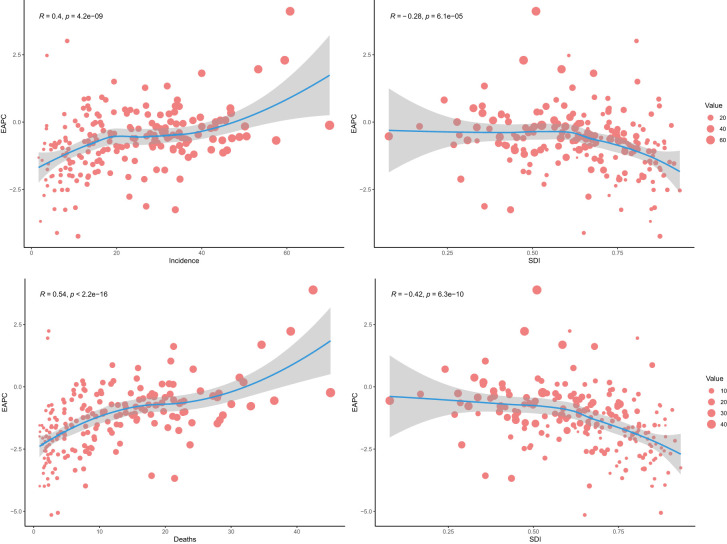
Relationship between the global estimated annual percentage change of age-standardized death rate and social-demographic index.

There was a moderate positive correlation between the EAPC of ASDR and ASDR (R: 0.54, *p*<0.001), suggesting that the ASDR increase in areas with increased ASDR (EAPC >0) further increased with the increase in ASDR, while the ASDR decrease in areas with decreased ASDR (EAPC <0) further decreased with the decrease in ASDR. Furthermore, there was a moderate negative correlation between the EAPC of ASDR and SDI (R: -0.42, *p*<0.001). The reduction in ASIR was significant with the SDI increase in areas with SDI >0.6 (**Figure 6**).

### Age

**Figure 7:**
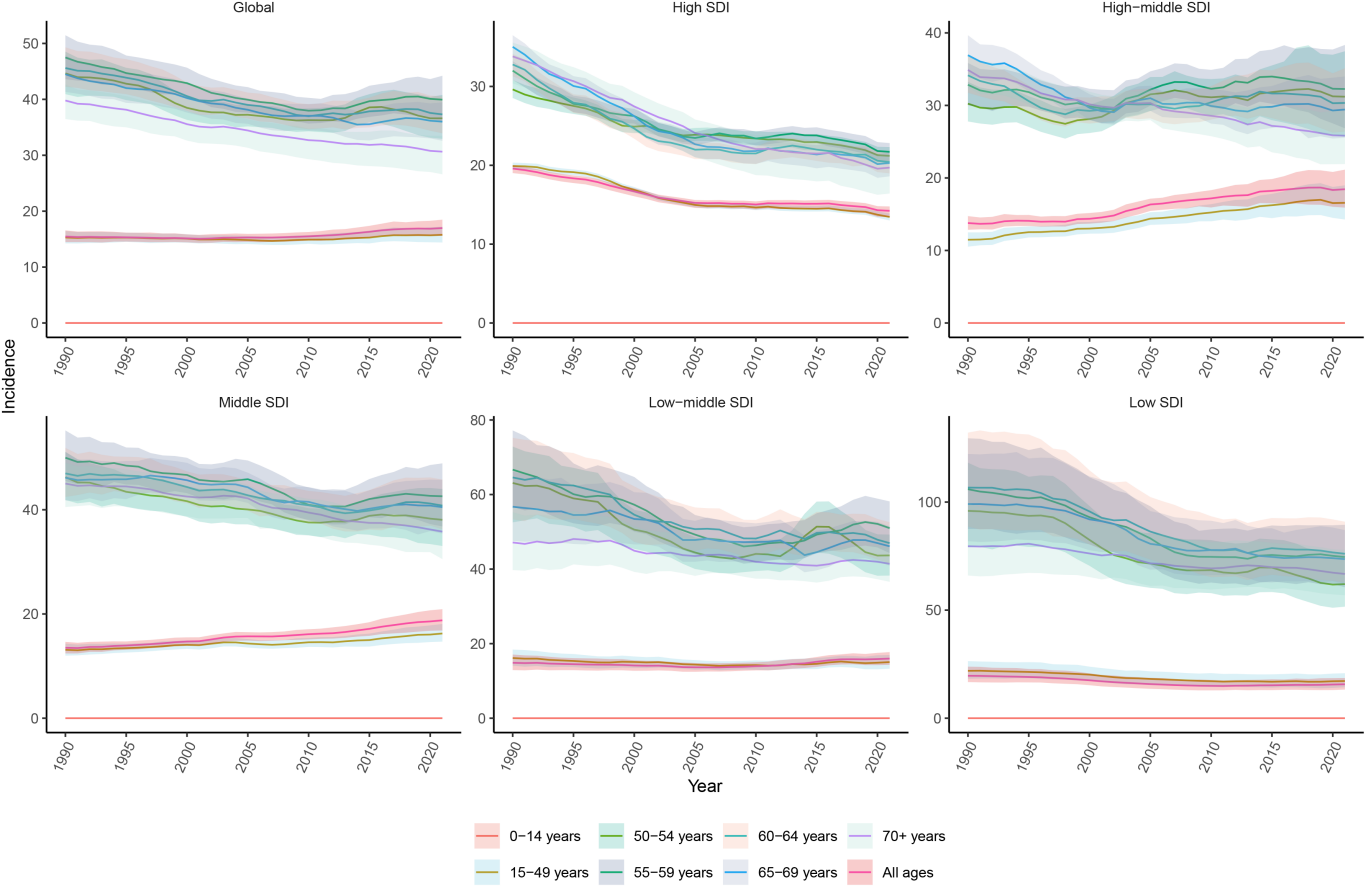
Age-specific trends in the incidence rate of cervical cancer from 1990 to 2021.

**Figure 8:**
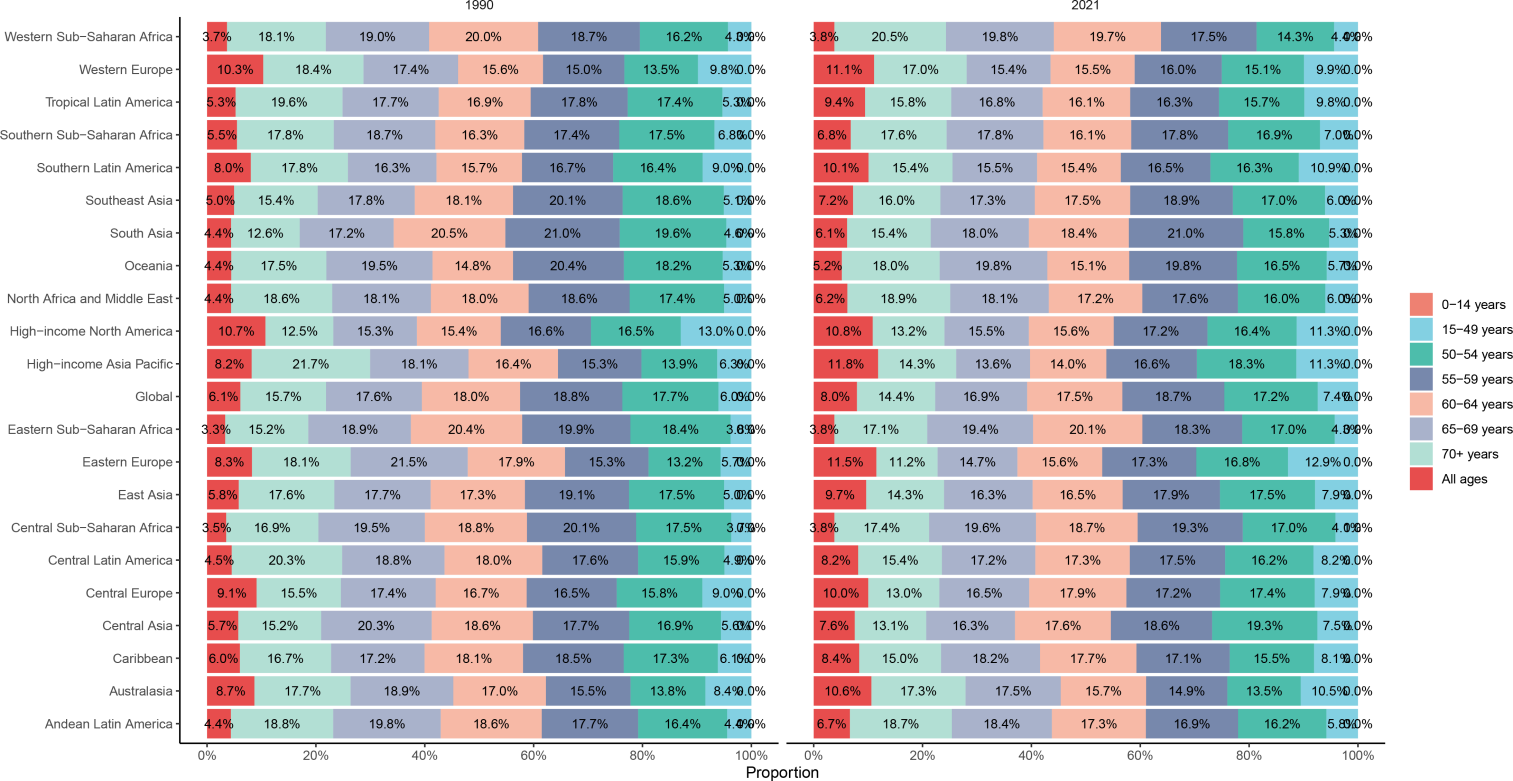
The proportions of age-specific cervical cancer incidence rates in 1990 and 2021.

In 2021, the highest and lowest global CC incidence was in the 55-59 and 15-49 (reproductive) age groups, respectively. However, the incidence did not show a clear age-dependent increasing trend, although the 50+ age group had a significantly higher incidence, when compared to the under 50 age group. In 2021, it was found that there was a slight increase in incidence in the 15-49 (childbearing) age group, and a decrease in incidence in the other age groups, when compared to 1990. In all five SDI regions, the CC incidence decreased in almost all age groups from 1990 to 2021. However, the medium and medium-high SDI regions presented with an increased incidence in the 15-49 (reproductive) age group. The low SDI region had the highest CC incidence rate among all five SDI regions, especially in the 60-64 age group. Overall, middle-aged and older women had significantly higher incidence rates, when compared to younger women of reproductive age. Furthermore, it was found that all SDI regions had the highest CC incidence rates in the 55-59 age group, except for the low SDI region, with the highest incidence rate observed in the 60-64 age group (**Figure 7**). In all 21 GBD regions, the 50-54, 55-59, 60-64, 65-69, and 70+ age groups comprised of the majority of the CC incidences (**Figure 8**).

The global CC death rate declined from 1990 to 2021 (**Supplementary Figure S3**). However, the death rate increased with aging, with the highest death rate identified in the 70+ group in all five SDI regions. The high SDI region had a lower death rate, when compared to the other SDI regions. However, the high SDI region had the largest reduction in death rate from 1990 to 2021, while the other SDI regions had smaller declines in death rates (**Supplementary Figure S10**). Among the 21 GBD regions, the 70+ group comprised the majority of CC death rates among all age groups (**Supplementary Figure S11**).

The DALY rates did not increase with aging, although the 55-59 group had the highest DALY rate. The DALY rates gradually declined in all five SDI regions from 1990 to 2021, with the greatest decline observed in the high SDI region (**Supplementary Figure S12**). This suggests that there were significant disparities in DALY rates among regions with different levels of socioeconomic development, although the overall global CC burden declined since 1990.

### Risk factors

Globally, behavioral risks are the only the contributors to death and DALYs. These behavioral risks mainly consisted of tobacco (smoke) and unsafe sex. Unsafe sex was the most significant contributor (reaching 100%) to CC death and DALYs in all SDI regions. Furthermore, unsafe sex was a necessary risk factor for death and DALYs in CC patients. Besides unsafe sex, smoke was the only risk factor for death and DALYs, although this was not a high risk factor in CC patients. Notably, higher SDI and smoke risk appeared to share the same region, suggesting that smoke might have more impact on death and DALYs in high SDI regions (**Supplementary Figures S13**, **S14**).

**Figure 9:**
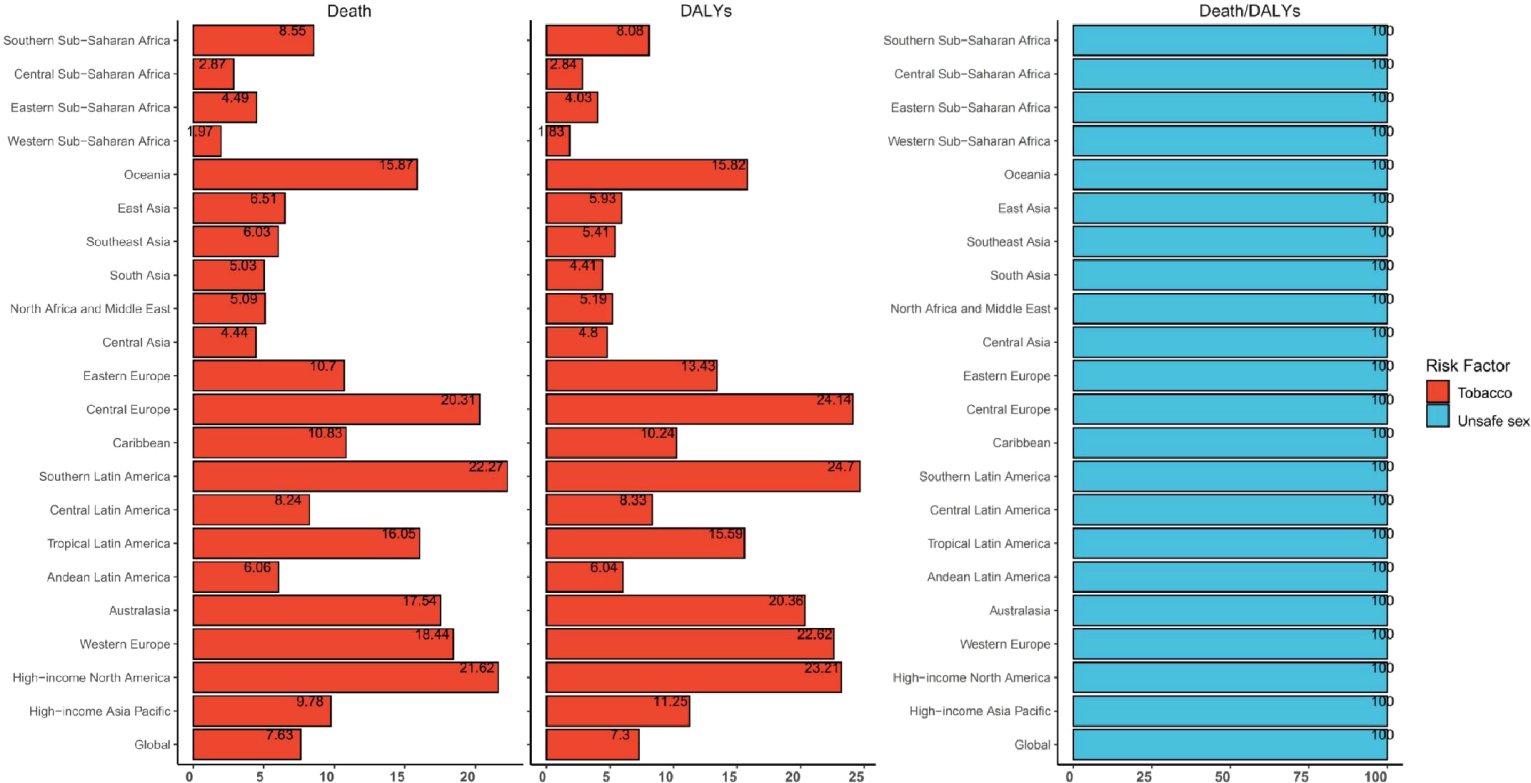
Global and regional cervical cancer disability-adjusted life-years and deaths attributable to risk factors in 2021.

Unsafe sex was the major contributor to CC death and DALYs in all 21 regions, which reached 100%. The region with smoke as the highest risk of death was Southern Latin America (22.27%), followed by high-income North America (21.62%) and Central Europe (20.31%). Furthermore, the region with smoke as the highest of DALY risk was Southern Latin America (24.7%), followed by Central Europe (24.14%) and high-income North America (23.21%) (**Figure 9**).

### Future forecasts of global burden of cervical cancer

**Figure 10:**
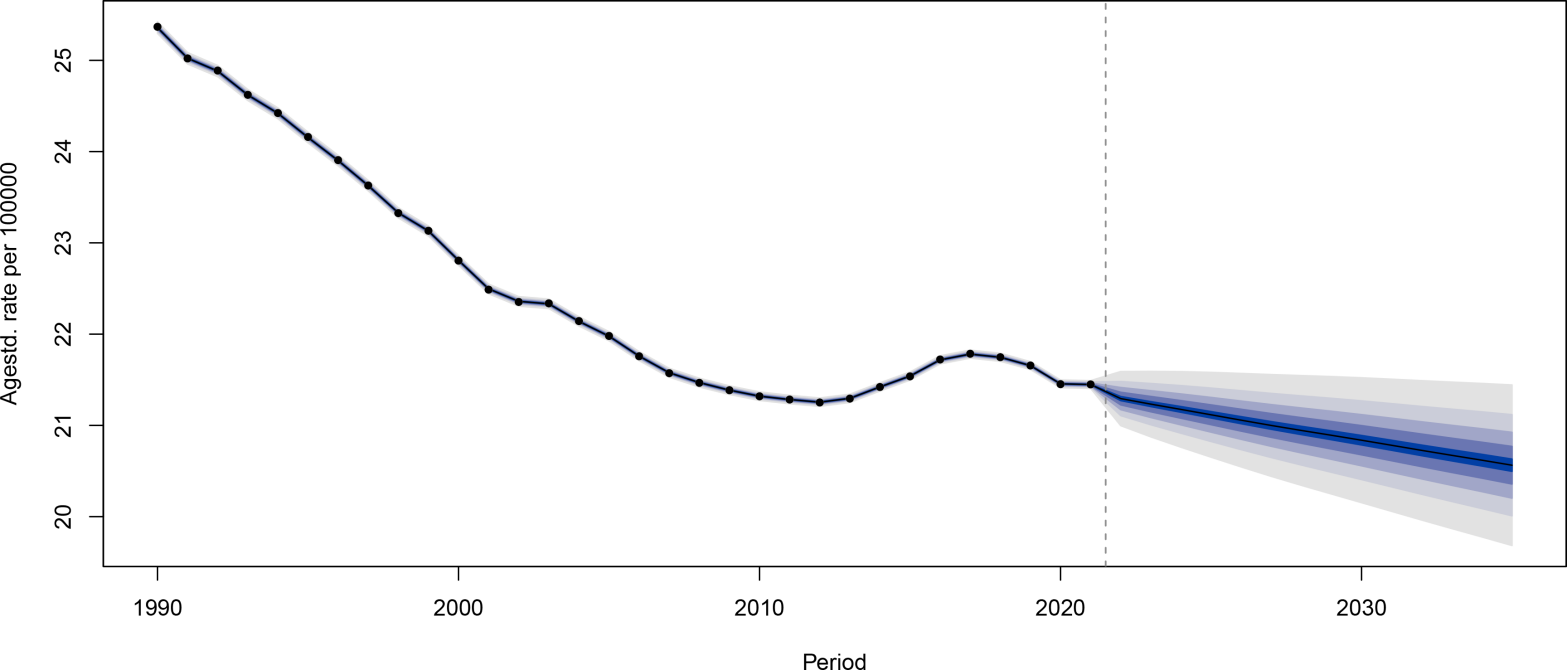
The global age-standardized incidence rates for cervical cancer from 1990 to 2021, with forecasted age-standardized incidence rate values projected from 2022 to 2035.

**Figure 11:**
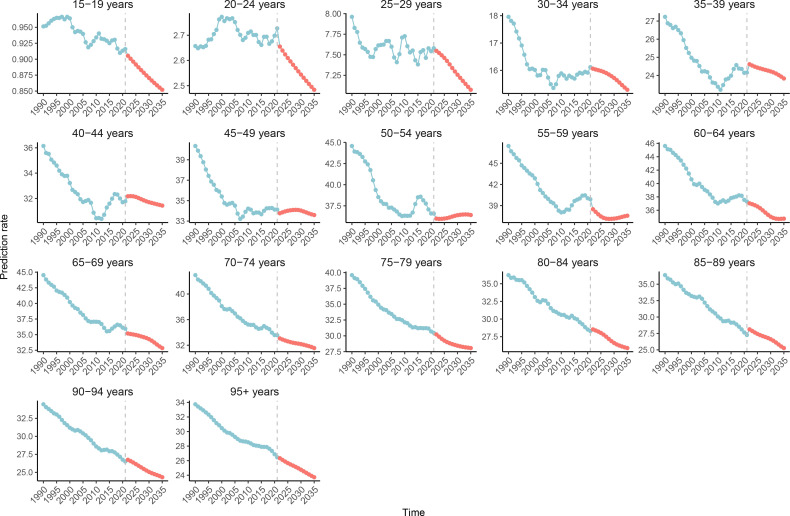
The global age specific age-standardized incidence rates for cervical cancer from 1990 to 2021, with forecasted age specific age-standardized incidence rate values projected for 2022 to 2035.

CC was projected to maintain a slow declining trend in ASIR globally from 2021 to 2035 (**Figure 10**). The ASIR decreased in all age groups, except for a small increase in the 50-54 age group (**Figure 11**). However, the global ASDR for CC significantly decreased over time (**Supplementary Figure S15**). Thus, ASDR significantly declined in all age groups (**Supplementary Figure S16**).

## Discussion

The present study analyzed the GBD 2021 database, and revealed that the overall trend in CC incidence rate and death rate decreased globally, although there were significant variations in CC burden across different SDI regions. CC ASIR slowly declined from 1990 to 2021, while there was a slight overall increase in CC incidence in middle and high development regions in the last 32 years. Although ASIR and ASDR exhibited a downward trend from 1990 to 2021, the number of incidences and deaths increased, underscoring the continuous efforts for CC control as a global public health problem.

Since the majority of CC cases were correlated to HPV infection, it was suggested that HPV vaccination coverage should be improved, especially by reducing regional disparities, in order to eradicate CC (26). The suboptimal reduction in ASIR in most of the regions might be correlated to the HPV vaccine coverage. Although a steady improvement in HPV vaccine uptake was observed in some countries, such as the United States, other countries continue to face a decrease or slow progress in HPV vaccine coverage (27). In the present study, the largest reduction in CC incidence was observed in Australasia (EAPC: -1.923, 95% CI: -2.188, -1.657), wherein the school-based program achieved 70-80% HPV vaccine coverage for the last dose in Australia (28). However, the high-income Asia-Pacific region had a somewhat elevated CC incidence from 2005 to 2021. In 2013, the Japanese government withdrew its unsolicited guidelines on HPV vaccines due to unconfirmed safety concerns, which might have contributed to the increasing trend of CC incidence in the region (29). Some countries, such as Italy, had a prominent increase in ASIR, which was probably due to low HPV vaccination rates (30).

A moderate positive correlation between EAPC, and ASIR and ASDR was observed. ASIR was the highest, but its reduction was greater in low SDI regions, suggesting that CC prevention and treatment significantly improved in low SDI regions, approaching the global average level. Furthermore, there was a moderate negative correlation between the reduction in ASDR and SDI, with the greatest reduction of ASDR in high SDI regions, suggesting a very successful CC prevention and treatment model in these regions. Thus, developing countries and regions should increase HPV vaccine coverage through strategies, such as more widely available low-cost vaccines, single-dose vaccination programs, vaccines with a long shelf life, better healthcare provider education, and the conduction of public awareness campaigns. In addition, HPV vaccine should still be recommended in women after hysterectomy due to high-grade cervical intraepithelial neoplasia or early-stage cervical cancer, since a recent study revealed that HPV vaccination can prevent the development of lower genital tract dysplasia in this patient population (31).

In areas with lower SDI and a potential for high risk of CC due to open sexual lifestyles, increased screening programs, better healthcare infrastructure, and increased awareness have led to a more correct CC diagnosis (11). The present study revealed that the decline in ASIR was significantly slower in areas with medium-high, medium, and medium-low SDI, which might be correlated to the aging population. CC remains as a prominent issue in Sub-Saharan Africa and Asia. Sub-Saharan Africa had a substantial increase in CC incidence from 1990 to 2021, which was the only region with a large increase in ASDR. In addition, East Asia and Eastern Europe were the only two regions, other than South Africa, that had a slight increase in CC incidence. China had the highest new incidence, and the second highest number of mortality from CC. Understanding the CC burden and risk factors in these countries would be critical for targeted prevention strategy development, rapid resource allocation, and the establishment of a comprehensive approach to address modifiable risk factors, which could improve its screening and early diagnosis, and ensure the equitable availability of quality healthcare in all SDI regions. In addition, specific intervention adjustments might be applied to certain circumstances in regions with distinct populations and development status, in order to counter the continuing high CC burden in Asia and Africa. In the present study, it was observed that there was a weak negative correlation between the EAPC of ASIR and SDI, and a moderate negative correlation between the EAPC of ASDR and SDI. This indicates that there is a lack of breakthrough in research for CC prevention and treatment, and that there is no better method in line with the present stage of the process to significantly reduce the ASDR of CC, although ASDR has been well-controlled in developed areas. In contrast, the reduction in ASDR in less developed regions significantly improved due to improvements in medical care and education. Therefore, there is a need to focus on the allocation of resources in less developed regions, and increase research on CC treatment, in order to eliminate CC at the global level, and improve the survival rate of CC cases. A recent study proposed different strategies for the surgical management of CC between high- and low-middle-income countries, in order to minimize its environmental impact, and reduce carbon output, providing a new area that is worthy of attention in CC management (32).

CC is the second most common cancer among women aged 15-44 years old (33). In the present study, it was found that the global risk of CC mortality steadily and rapidly increased with age, indicating that CC is not just a disease of young women. The increasing risk of death with age might reflect the accumulation of biological risk factors in the body, especially HPV infection (34). Therefore, the focus of the present study was on middle-aged and older women, who had significantly higher ASIR and ASDR, when compared to younger and childbearing age women, with a peak ASIR in the 55-59 age group, a peak ASDR in the 70+ age group, and a peak age-standardized DALY in the 55-59 age group. In future clinical practice, more prevention and treatment strategies that target women in the above age groups should be developed and implemented. With the increasing number of CC cases in middle-aged and older women, it is important that gynecologists are appropriately trained and educated on the CC screening, diagnosis, treatment and follow-up of these women. This is essential, because the accuracy of the CC screening depends not only on the screening itself, but also on the correct performance of the Pap smear or colposcopy, treatment, and follow-ups. In addition, older women are more likely to have comorbidities with other systemic diseases, reducing their chance to surgery. Therefore, simultaneous treatments, such as radiotherapy, should be developed and popularized as an alternative means of radical treatment, in order to improve the survival of CC patients, especially for non-surgical cervical cancer. Novel diagnostic approaches, such as positron emission tomography-magnetic resonance imaging, and new treatment methods, such as minimally invasive pelvic exenteration, conization prior to radical hysterectomy, and targeted immune and radiation therapy, should be developed to provide accurate clinical staging in CC, and improve patient survival, while minimizing complications (35–37). Furthermore, CC diagnosis and treatment guidelines have significant regional variations. Thus, international cooperations and multicenter studies are required to determine whether the differences are due to different patient characteristics or study methods, and determine whether the differences impact patient management (38). CC patients can develop decreased sexual function and quality of life after treatment, suggesting that preoperative planning and postoperative care should consider these complications (39).

The present analysis of risk factors revealed that unsafe sex was the most prominent contributor to CC DALYs and death globally, followed by smoke. Unsafe sex was a necessity for CC DALYs and death (40). Although the proportion of CC DALYs and deaths attributable to smoke exhibited a decreasing trend from 1990 to 2021, the attributable risk of death still reached 17.05% and 11.27%, respectively, and the attributable risk of DALYs reached 19.29% and 12.19%, respectively, in high and medium-high SDI regions. Therefore, it is important to pay attention to modifiable risk factors in CC prevention strategies. The different risk factor attribution ratios across SDI regions necessitate tailored interventions that consider local circumstances and resources.

The present study presented the first projections of global ASIR and ASDR trends for CC, as well as the first projections of CC incidence and death rate trends by age group. Overall, CC will continue to be an important cause of morbidity and mortality for women globally in the future, with significantly lower incidence rates in younger women, when compared to the previous period. This is correlated to the wide promotion and vaccination of HPV vaccines, the slower decrease in ASIR, and a more pronounced decrease in ASDR in older women, which in turn, is correlated to global aging, the increase in screening and diagnostic levels, and the upgrading of treatment options on surgery and chemoradiation therapy. Notably, the incidence of CC appeared to be increasing in some middle-aged women. Special attention should be given in the future to women in the age group of 50-54 years old, with the necessity to advance HPV-based screening programs for early intervention and treatment in high-risk groups. The projection of the future disease burden of CC in the present study emphasized the continuing challenge of eradicating CC globally, and the necessity of sustained efforts in its prevention, early detection, and treatment.

The present study provided a comprehensive analysis on the global and regional burden of CC, and its related attributable risk factors, as well as the future projected trend. However, the present study had several limitations. The analysis was based on the GBD study 2021. The quality and availability of data could significantly vary, leading to the potential underestimation or overestimation of disease burden. Furthermore, although the potential attributable risk factors to CC were analyzed, a number of other factors, such as genetic predisposition, unhealthy lifestyle and behaviors (smoking, and diets that are high in saturated fat and trans fat), environmental exposure, weakened immune system (human immunodeficiency virus infection), and concurrent sexually transmitted diseases, could also be correlated to CC development. These data were not included in the GBD study 2021, which might result in the inadequate estimation of the full spectrum of CC risk factors and its contributions to disease burden. Moreover, although a projection for future CC burden was provided, this projection was based on present CC trends and patterns, which could certainly be affected by risk factor change, screening technology, and local healthcare policy shifts.

## Conclusion

There was a global, regional and national downward trend in ASIRs, ASDRs, and age-standardized DALYs for CC from 1990 to 2021. This declining trend might persist until 2035. Furthermore, there were significant differences in CC incidence, mortality, and disability among regions with different socioeconomic development, as well as among patients with different ages and risk factors. CC remains as a major public health issue. The local government and authorities should develop effective prevention and management strategies, including improved access to healthcare in economically less developed areas, targeted screening and preventive measures, and risk factor modifications. Specifically, HPV vaccines should be advocated and offered to eligible women. Furthermore, adequate pre- and post-treatment care should be provided to CC women. Meanwhile, more research is required, with focus on the development of cost-effective and environmentally friendly strategies adapted to local socioeconomic development and resources, in order to minimize the disparity in preventive measures, reduce the global burden of CC, mitigate climate change, and improve overall health. The outcomes for the implementation of preventive strategies and interventional measures should be examined in different SDI backgrounds. In addition, CC data collection and quality monitoring in resource-poor areas should be improved, in order to more accurately understand the CC trends and intervention effectiveness.

## Data Availability

Publicly available datasets were analyzed in this study. This data can be found here: The Global Burden of Disease study 2021 data resources are available online from the Global Health Data Exchange (GHDx) query tool (http://ghdx.healthdata.org/gbd-results-tool).

## Supplementary material

Supplementary Figure 1 The global disease burden of cervical cancer age-standardized death rates for the 204 countries and territories.

Supplementary Figure 2 The global disease burden of cervical cancer estimated annual percentage changes in age-standardized death rates for the 204 countries and territories.

Supplementary Figure 3 Trends for the cervical cancer age-standardized death rates from 1990 to 2021.

Supplementary Figure 4 The global disease burden of cervical cancer age-standardized disability-adjusted life-year rates for the 204 countries and territories.

Supplementary Figure 5 The global disease burden of cervical cancer estimated annual percentage changes in age-standardized disability-adjusted life-year rates for the 204 countries and territories.

Supplementary Figure 6 Trends for the cervical cancer age-standardized DALY rates from 1990 to 2021.

Supplementary Figure 7 The relationship between the age-standardized death rates and social-demographic index in the 21 regions from 1990 to 2021.

Supplementary Figure 8 The relationship between the age-standardized disability-adjusted life-year rates and social-demographic index in the 21 regions from 1990 to 2021.

Supplementary Figure 9 The relationship between the age-standardized death rates and social-demographic index in the 204 countries and territories from 1990 to 2021.

Supplementary Figure 10 Age-specific trends in death rate for cervical cancer.

Supplementary Figure 11 The proportion of age-specific cervical cancer death rates in 1990 and 2021.

Supplementary Figure 12 Age-specific trends for the disability-adjusted life-year rates of cervical cancer.

Supplementary Figure 13 Proportion of deaths attributable to risk factors for the five social-demographic index regions in 2021.

Supplementary Figure 14 Proportion of disability-adjusted life-years attributable to risk factors for the five social-demographic index regions in 2021.

Supplementary Figure 15 The age-standardized death rates for cervical cancer was assessed globally from 1990 to 2021, with forecasted age-standardized death rate values projected for 2022 to 2035.

Supplementary Figure 16 The age specific age-standardized death rates for cervical cancer were assessed globally from 1990 to 2021, with forecasted age specific age-standardized death rate values projected for 2022 to 2035.
