# Supplementary figures and images for: Cervical cancer burden and attributable risk factors across different age and regions from 1990 to 2021 and future burden prediction: results from the global burden of disease study 2021
Source: Front Oncol. 2025 Feb 7;15:1541452. doi: 10.3389/fonc.2025.1541452 (PMC11842224; doi:10.3389/fonc.2025.1541452)

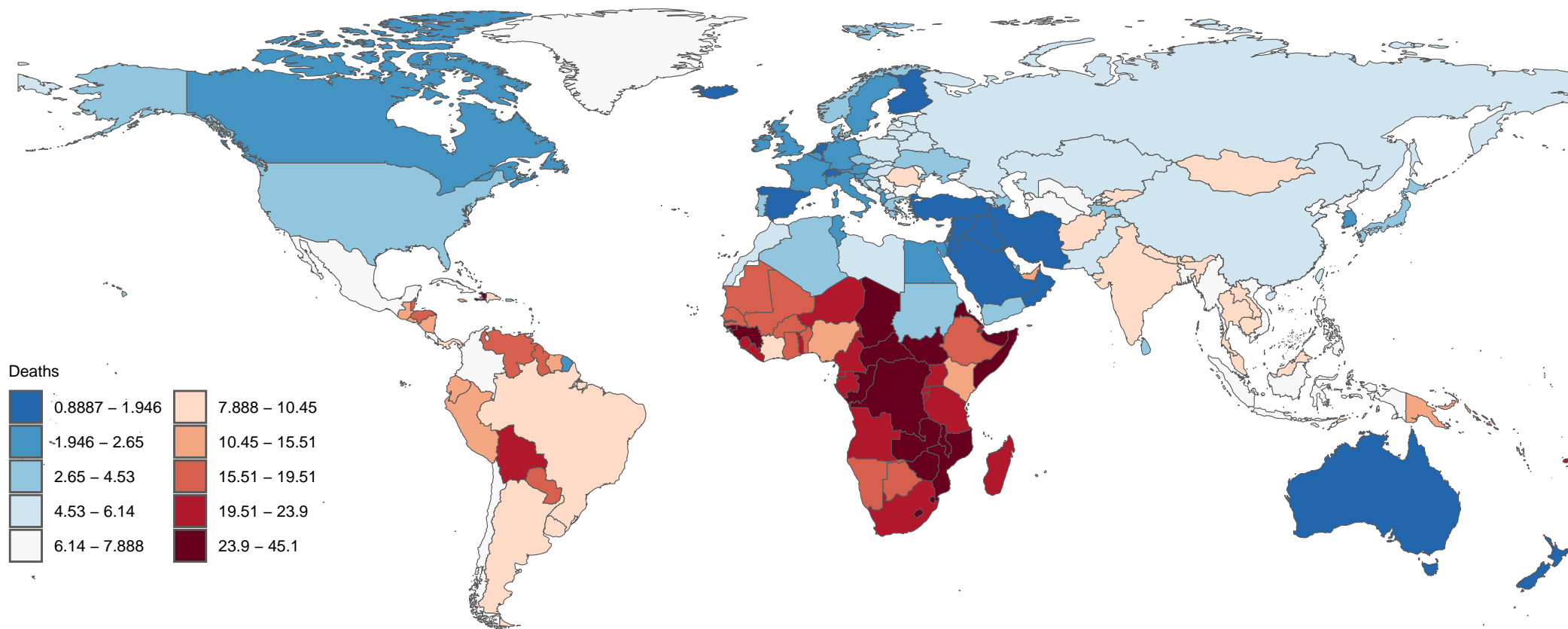

Supplement: Supplementary Figure 1 — The global disease burden of cervical cancer age-standardized death rates for the 204 countries and territories. [file DataSheet1.zip › Supplementary Figures/Fig. S1.pdf]

Global

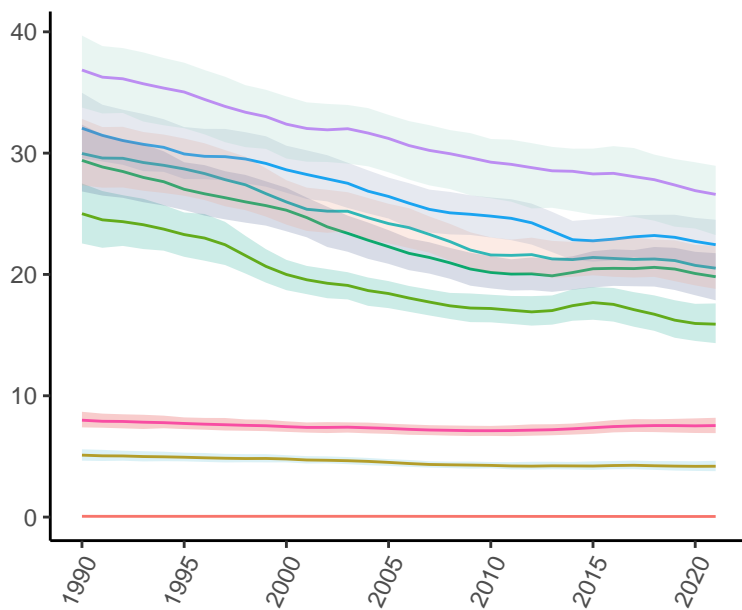

High SDI

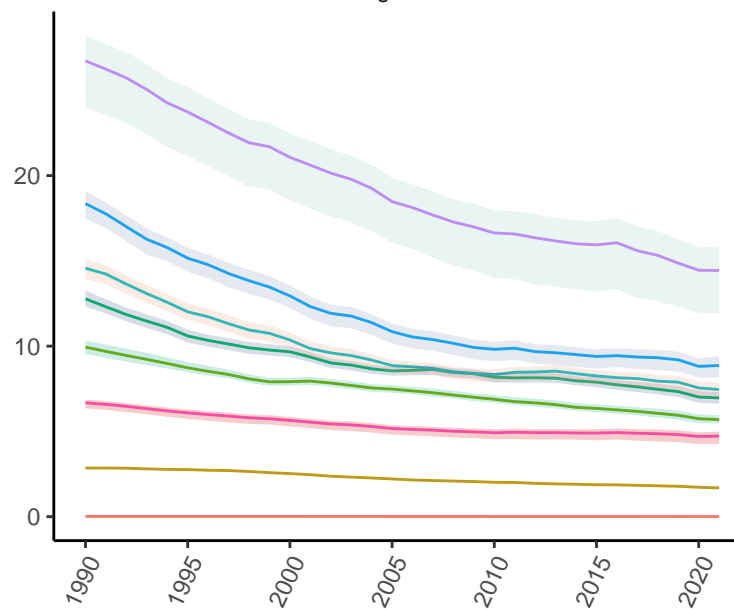

High-middle SDI

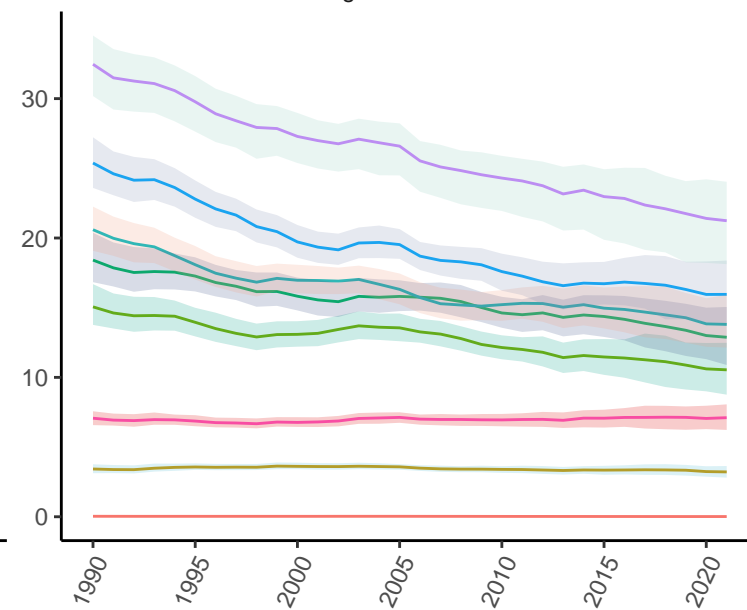

Middle SDI

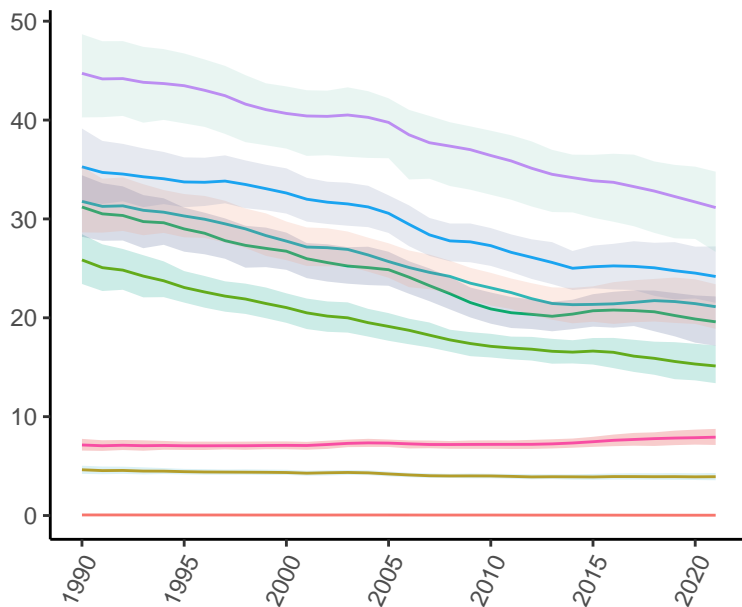

Low-middle SDI

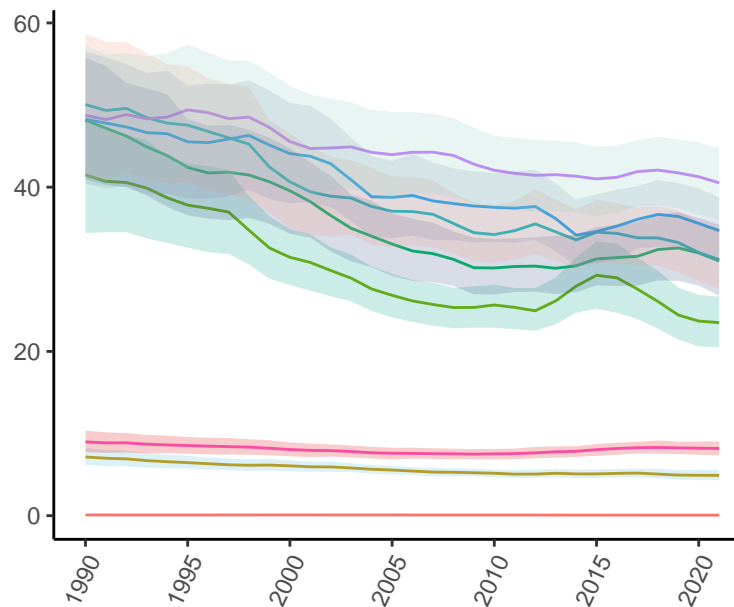

Low SDI

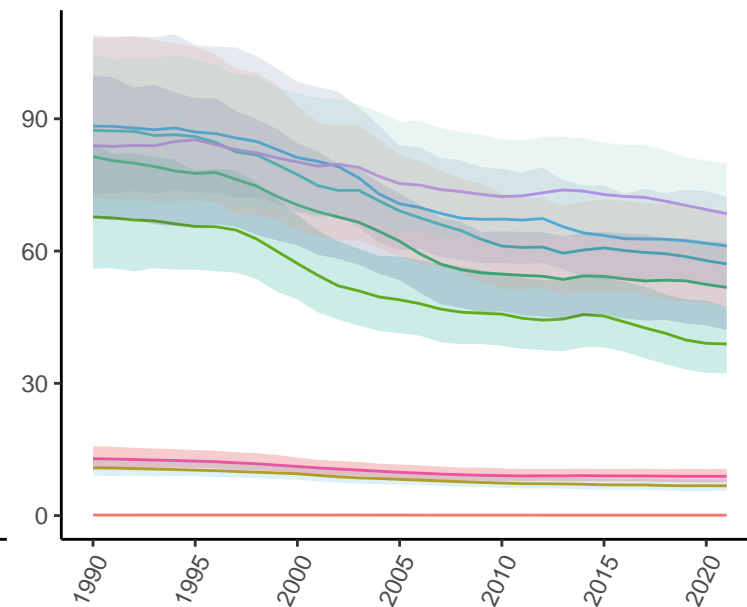

Year

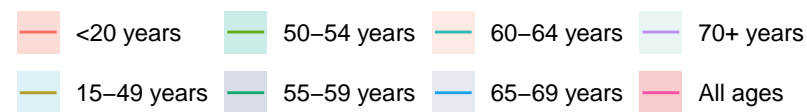

Supplement: Supplementary Figure 1 — The global disease burden of cervical cancer age-standardized death rates for the 204 countries and territories. [file DataSheet1.zip › Supplementary Figures/Fig. S10.pdf]

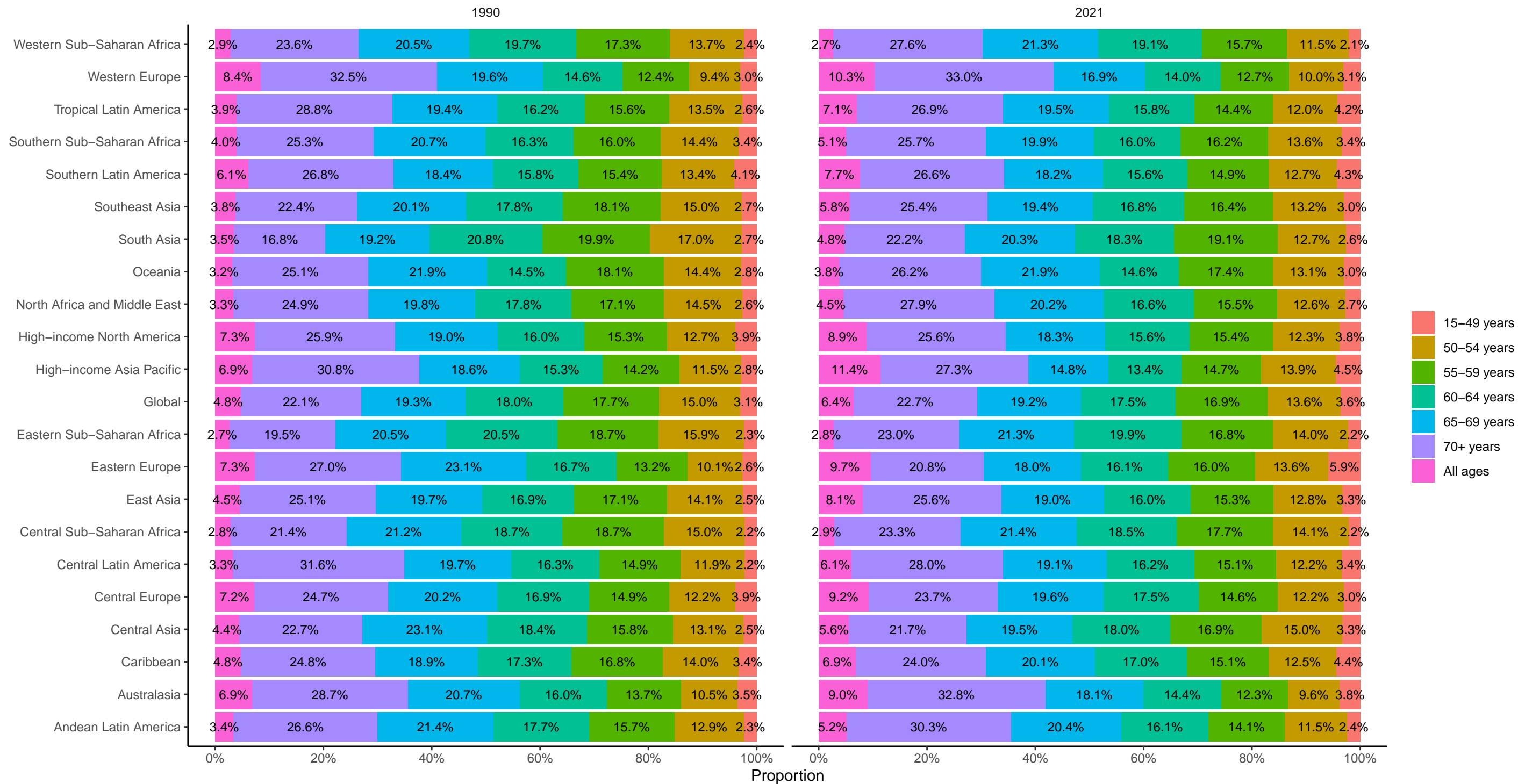

Supplement: Supplementary Figure 1 — The global disease burden of cervical cancer age-standardized death rates for the 204 countries and territories. [file DataSheet1.zip › Supplementary Figures/Fig. S11.pdf]

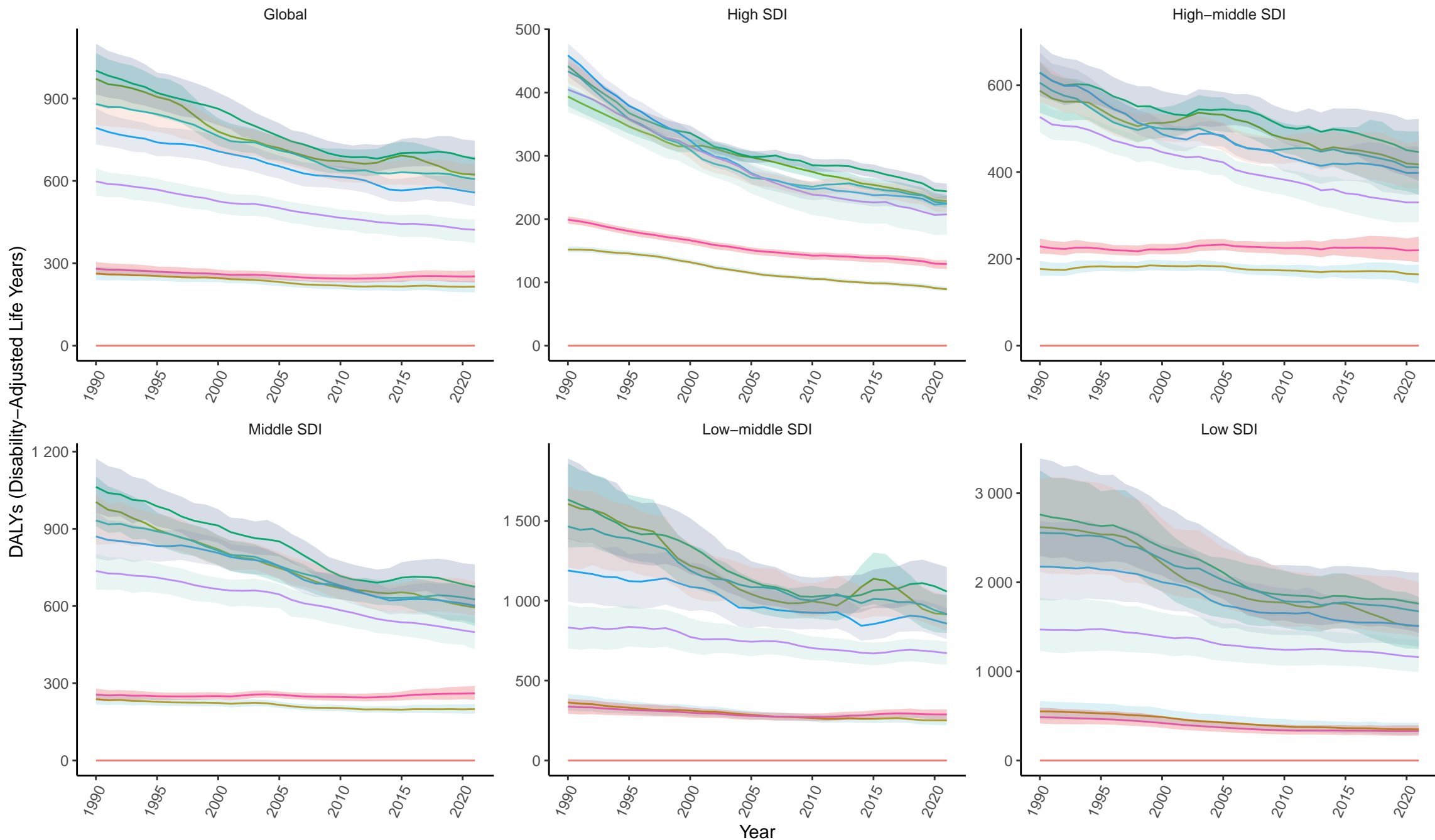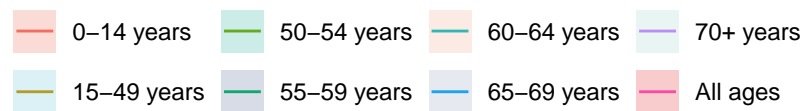

Supplement: Supplementary Figure 1 — The global disease burden of cervical cancer age-standardized death rates for the 204 countries and territories. [file DataSheet1.zip › Supplementary Figures/Fig. S12.pdf]

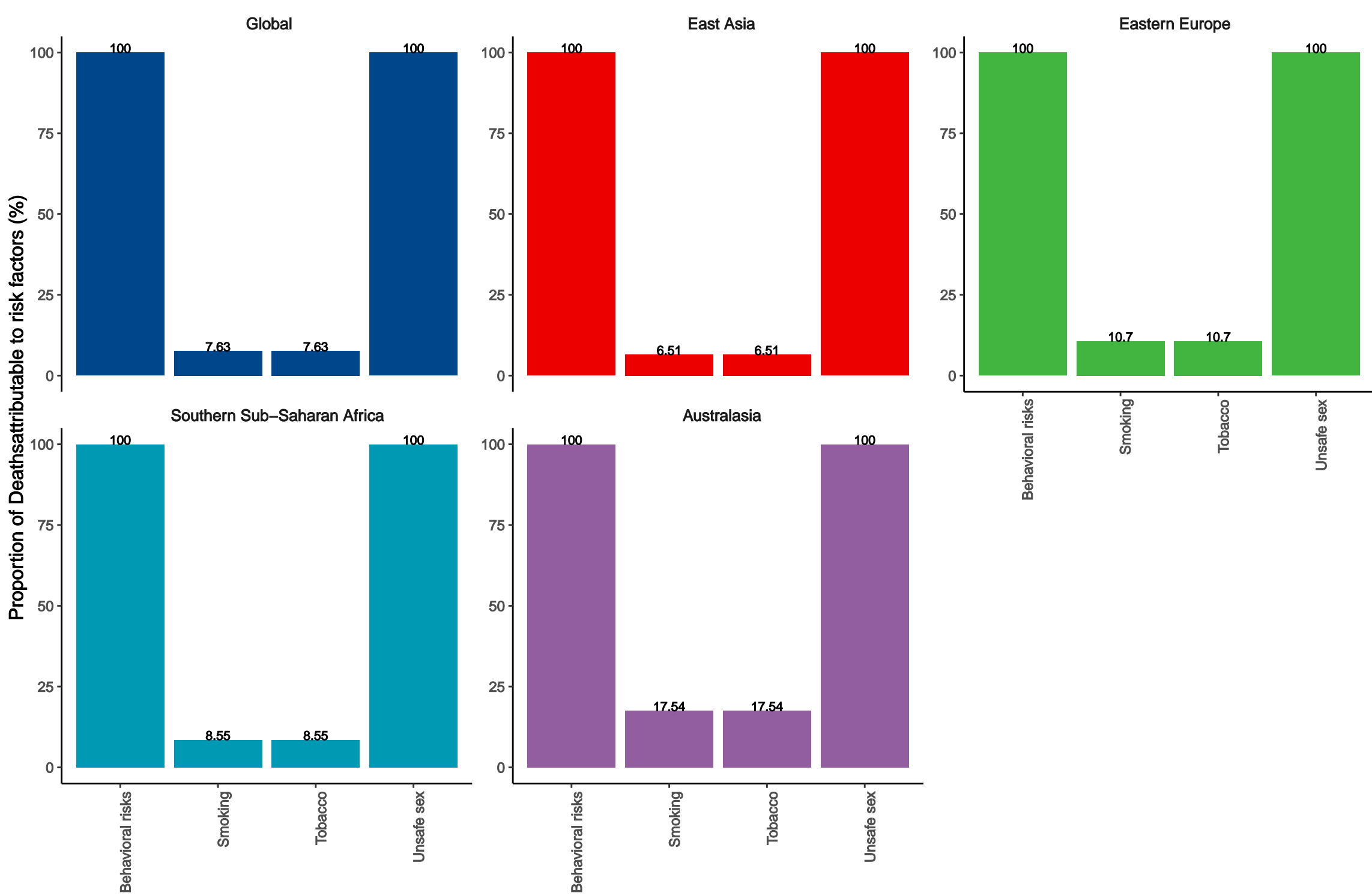

Supplement: Supplementary Figure 1 — The global disease burden of cervical cancer age-standardized death rates for the 204 countries and territories. [file DataSheet1.zip › Supplementary Figures/Fig. S13.pdf]

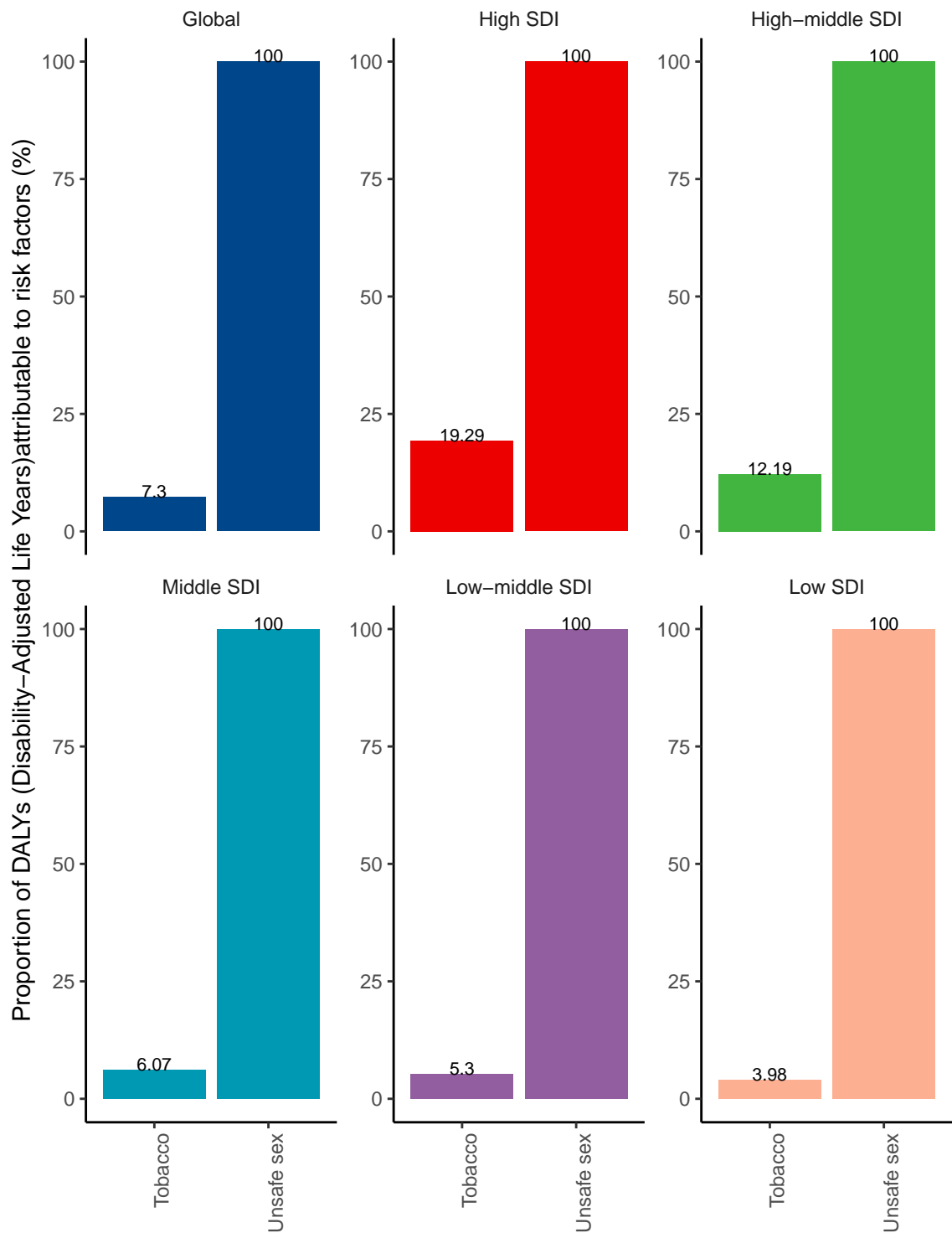

Supplement: Supplementary Figure 1 — The global disease burden of cervical cancer age-standardized death rates for the 204 countries and territories. [file DataSheet1.zip › Supplementary Figures/Fig. S14.pdf]

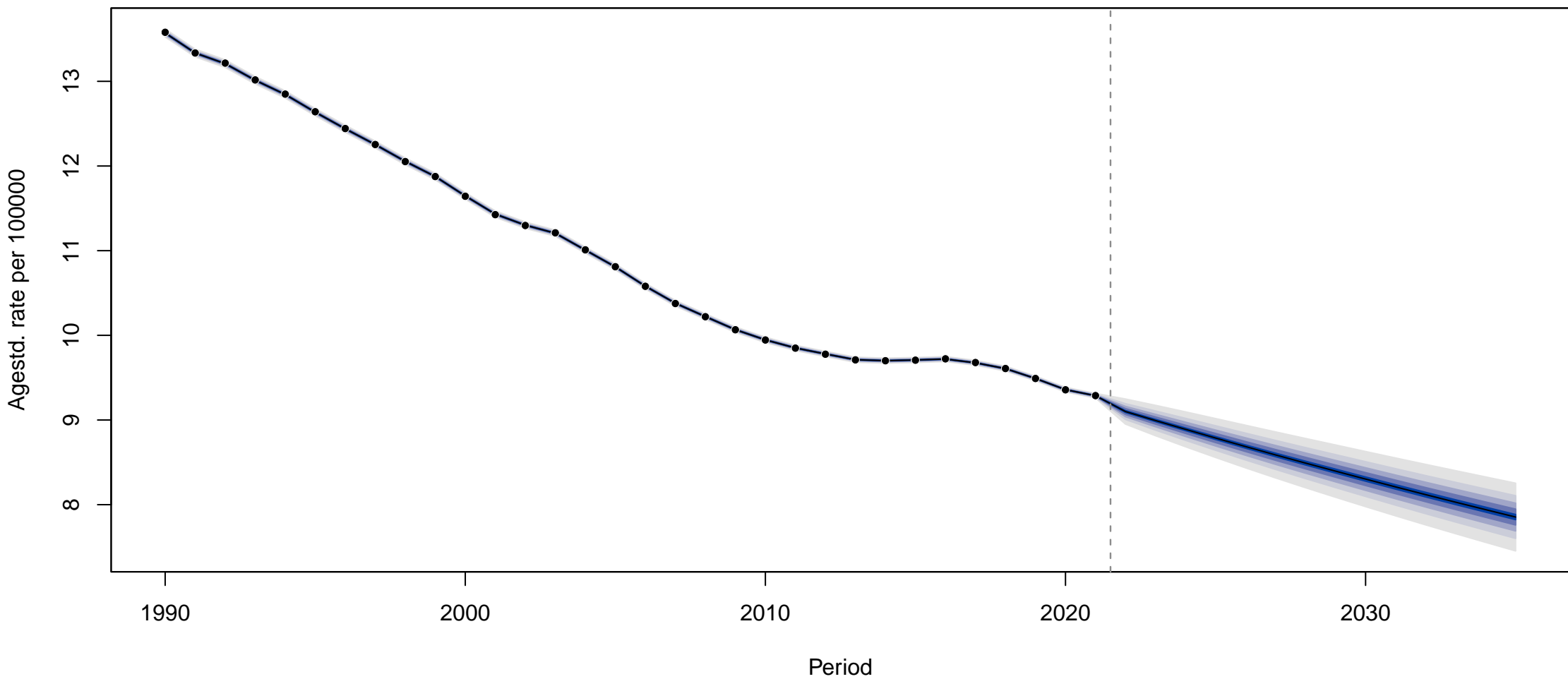

Supplement: Supplementary Figure 1 — The global disease burden of cervical cancer age-standardized death rates for the 204 countries and territories. [file DataSheet1.zip › Supplementary Figures/Fig. S15.pdf]

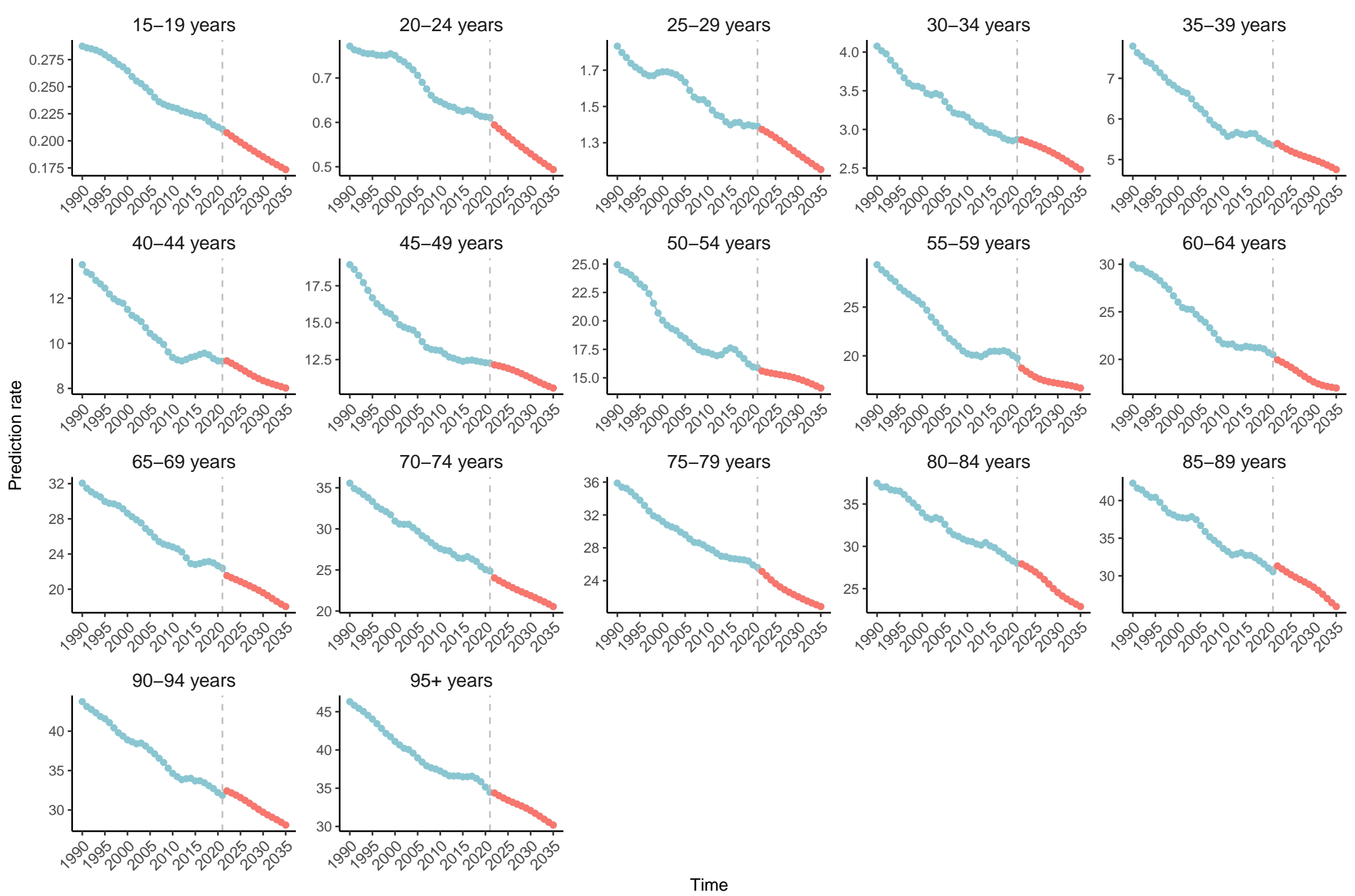

Supplement: Supplementary Figure 1 — The global disease burden of cervical cancer age-standardized death rates for the 204 countries and territories. [file DataSheet1.zip › Supplementary Figures/Fig. S16.pdf]

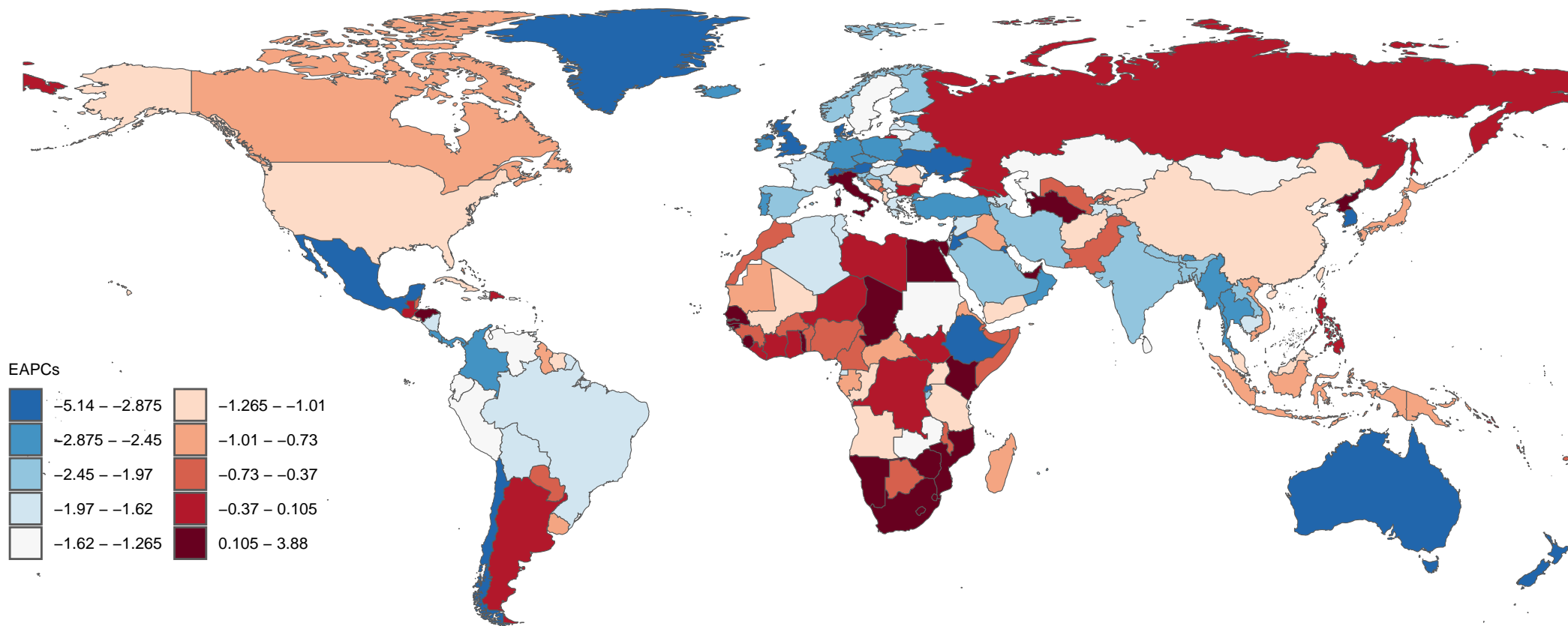

Supplement: Supplementary Figure 1 — The global disease burden of cervical cancer age-standardized death rates for the 204 countries and territories. [file DataSheet1.zip › Supplementary Figures/Fig. S2.pdf]

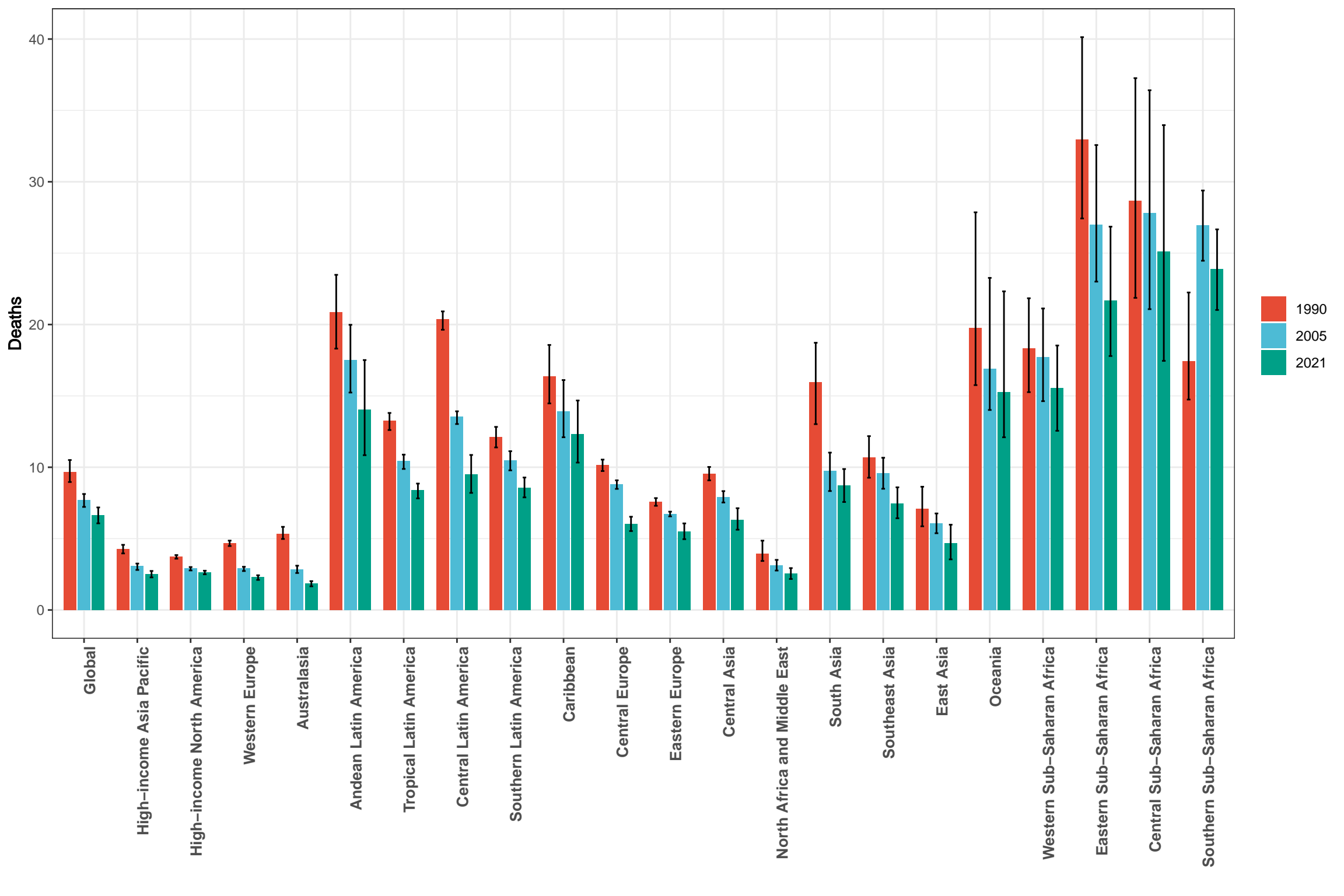

Supplement: Supplementary Figure 1 — The global disease burden of cervical cancer age-standardized death rates for the 204 countries and territories. [file DataSheet1.zip › Supplementary Figures/Fig. S3.pdf]

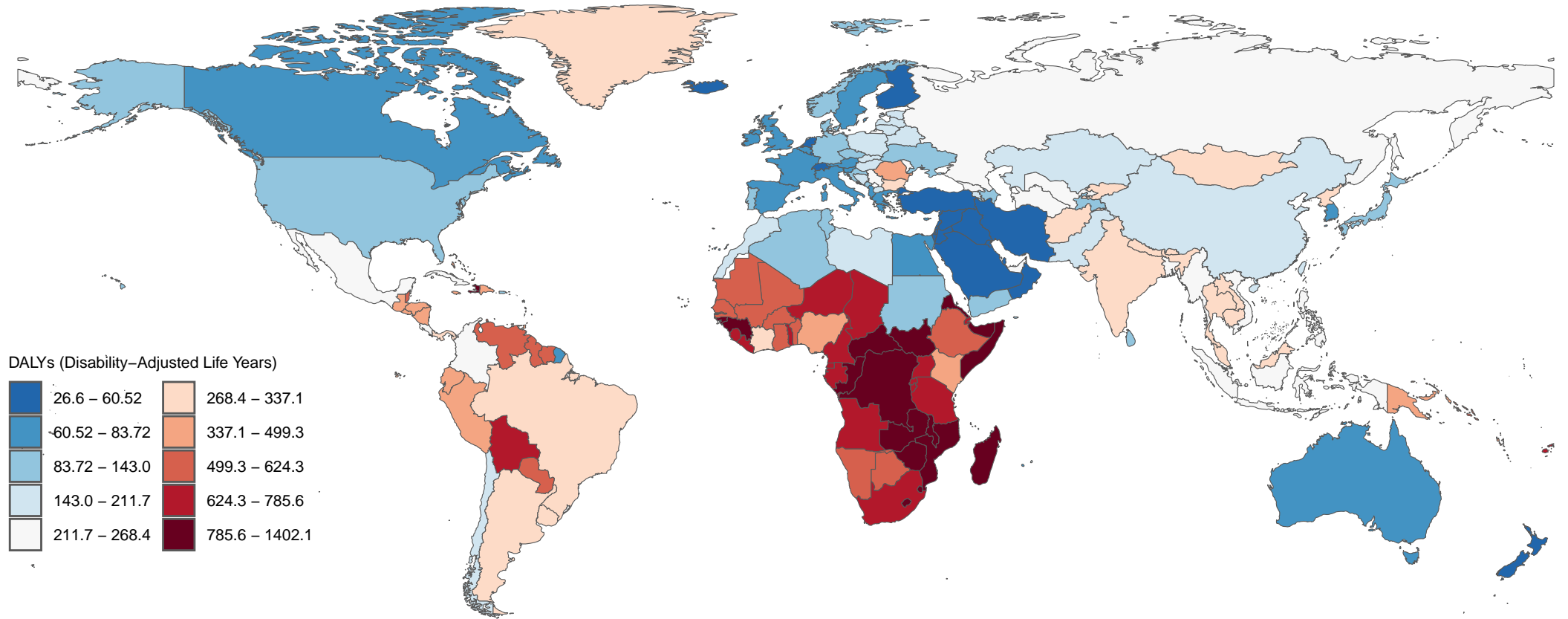

Supplement: Supplementary Figure 1 — The global disease burden of cervical cancer age-standardized death rates for the 204 countries and territories. [file DataSheet1.zip › Supplementary Figures/Fig. S4.pdf]

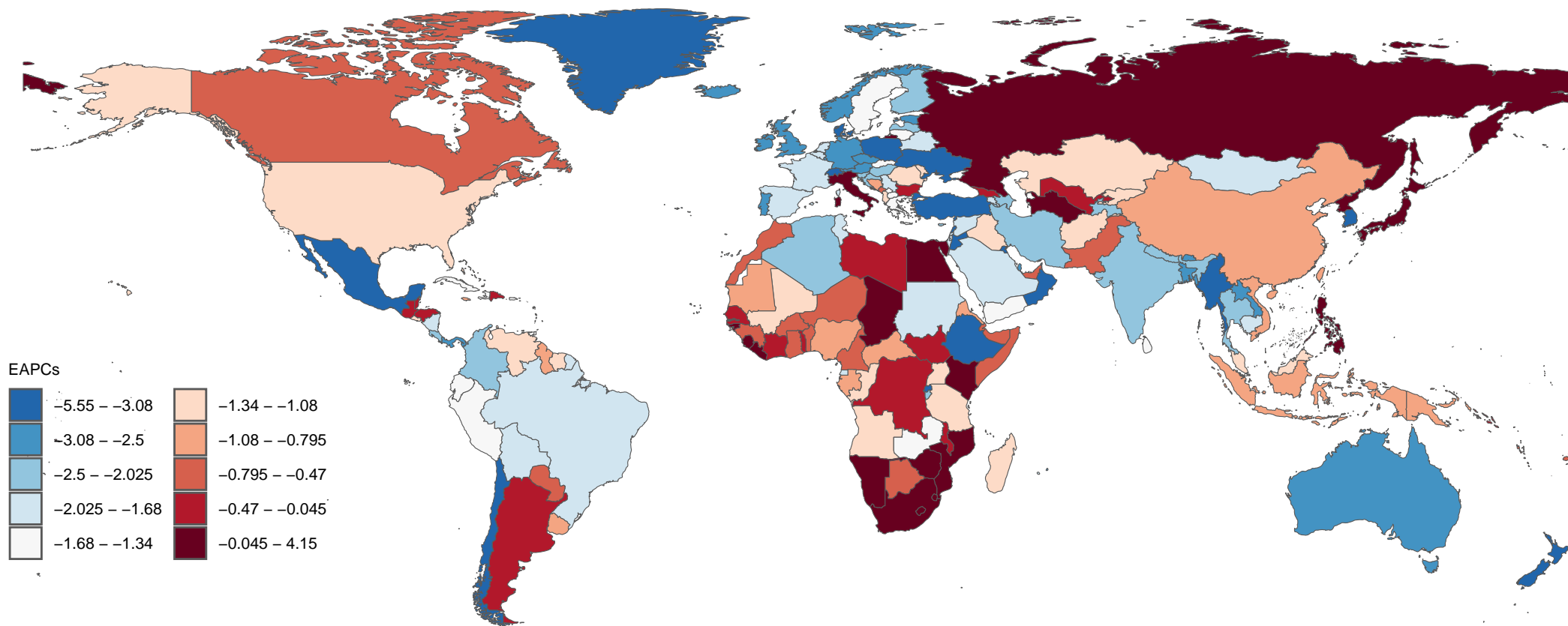

Supplement: Supplementary Figure 1 — The global disease burden of cervical cancer age-standardized death rates for the 204 countries and territories. [file DataSheet1.zip › Supplementary Figures/Fig. S5.pdf]

DALYs (Disability-Adjusted Life Years)

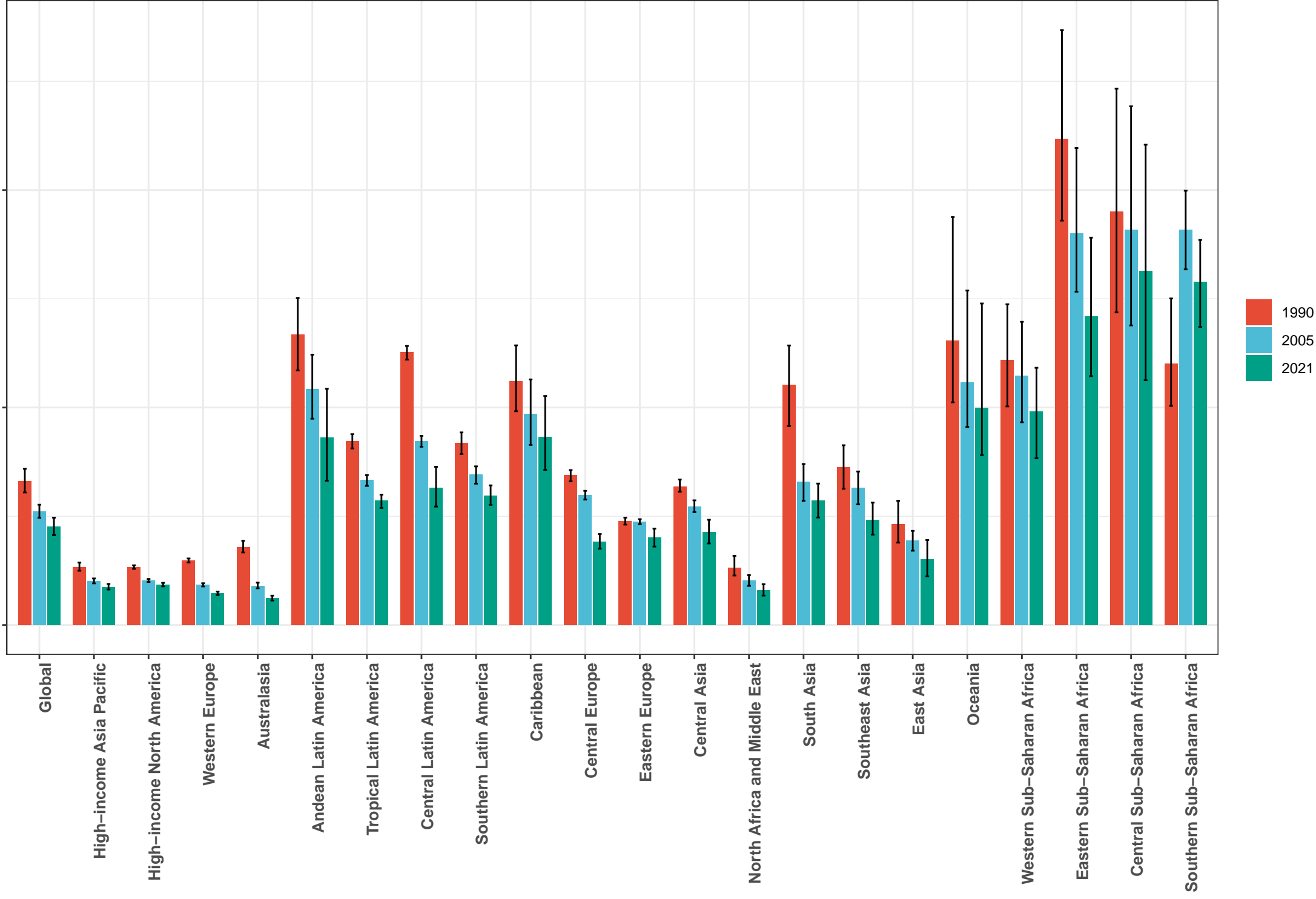

Supplement: Supplementary Figure 1 — The global disease burden of cervical cancer age-standardized death rates for the 204 countries and territories. [file DataSheet1.zip › Supplementary Figures/Fig. S6.pdf]

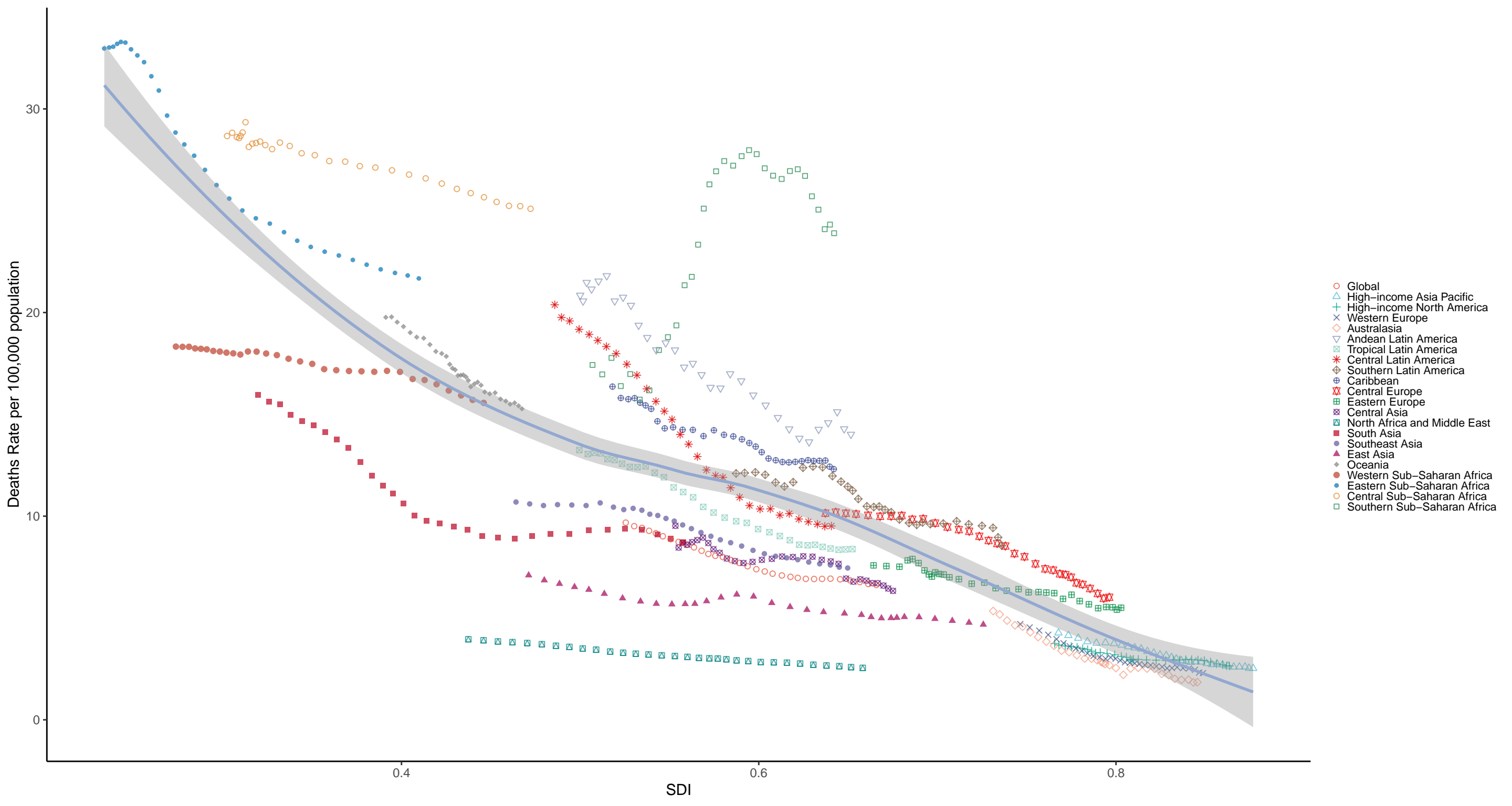

Supplement: Supplementary Figure 1 — The global disease burden of cervical cancer age-standardized death rates for the 204 countries and territories. [file DataSheet1.zip › Supplementary Figures/Fig. S7.pdf]

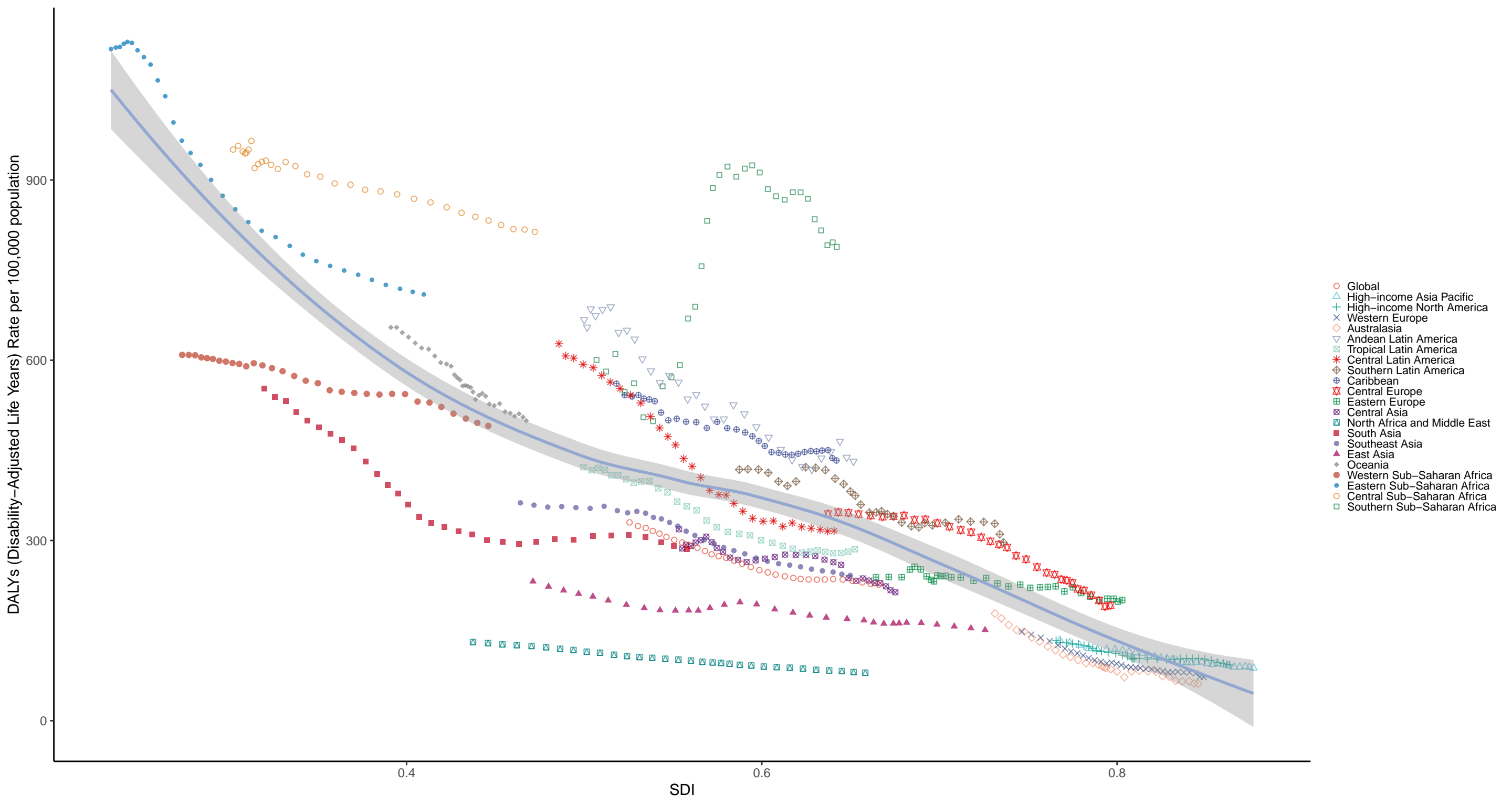

Supplement: Supplementary Figure 1 — The global disease burden of cervical cancer age-standardized death rates for the 204 countries and territories. [file DataSheet1.zip › Supplementary Figures/Fig. S8.pdf]

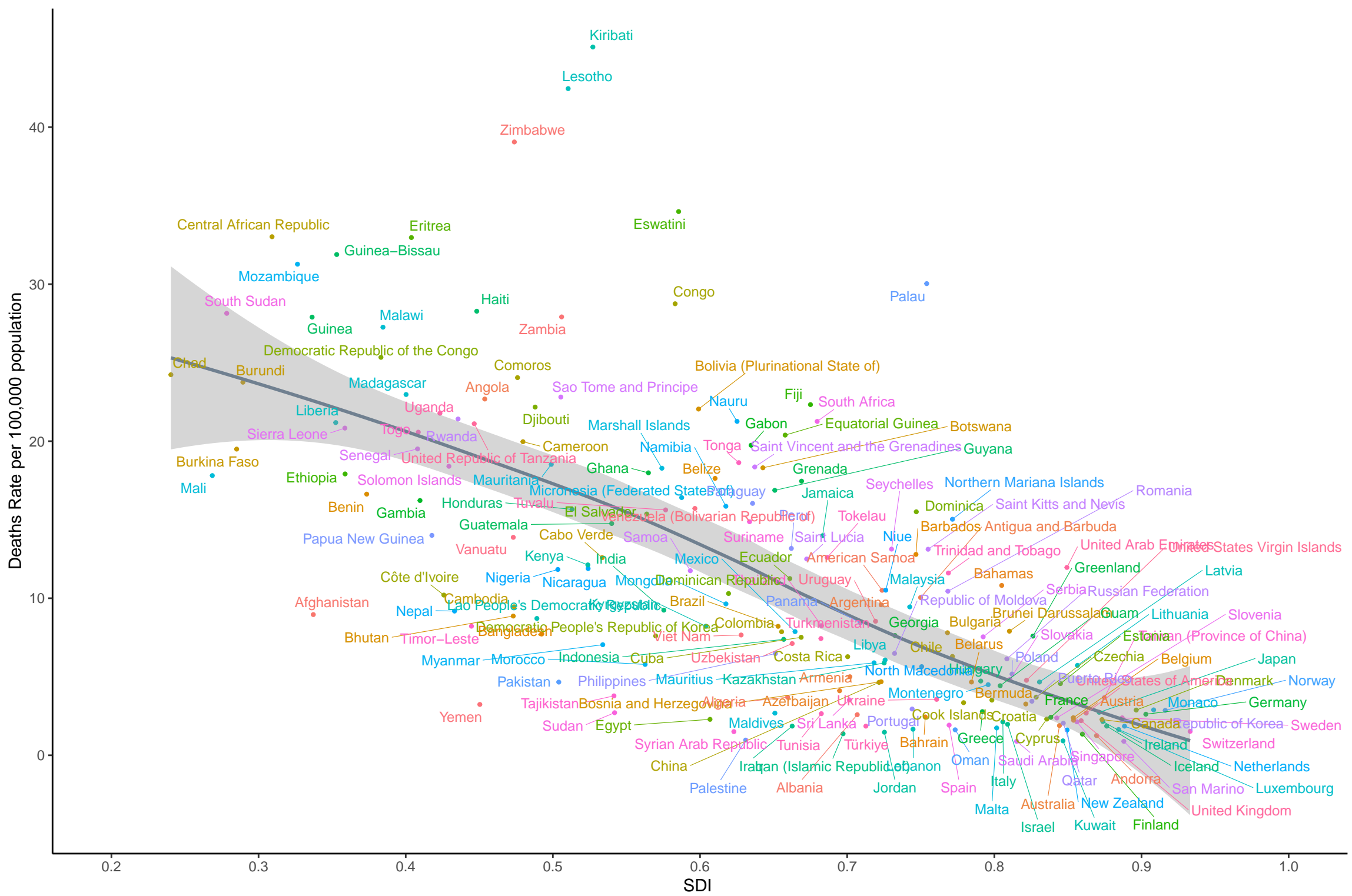

Supplement: Supplementary Figure 1 — The global disease burden of cervical cancer age-standardized death rates for the 204 countries and territories. [file DataSheet1.zip › Supplementary Figures/Fig. S9.pdf]
