# Supplementary material for: Cervical cancer burden and attributable risk factors across different age and regions from 1990 to 2021 and future burden prediction: results from the global burden of disease study 2021
Source: Front Oncol. 2025 Feb 7;15:1541452. doi: 10.3389/fonc.2025.1541452 (PMC11842224; doi:10.3389/fonc.2025.1541452)
Supplement: Supplementary file 2 [file DataSheet2.pdf]

GBD 2021Geographies

Super regions	Regions	Country names	
**Central Europe, Eastern Europe, and Central Asia**			
	**Central Asia**		
		Armenia	
		Azerbaijan	
		Georgia	
		Kazakhstan	
		Kyrgyzstan	
		Mongolia	
		Tajikistan	
		Turkmenistan	
		Uzbekistan	
	**Central Europe**		
		Albania	
		Bosnia and Herzegovina	
		Bosnia and Herzegovina	
		Croatia	
		Czech Republic	
		Hungary	
		Macedonia	
		Montenegro	
		Poland	
		Romania	
		Serbia	
		Slovakia	
		Slovenia	
	**Eastern Europe**		
		Belarus	
		Estonia	
		Latvia	
		Lithuania	
		Moldova	
		Russia	
		Ukraine	
**High-income**			
	**Australasia**		
		Australia	
		New Zealand	
	**High-income Asia Pacific**		
		Brunei	
		Japan	
		Singapore	
		South Korea	
	**High-income North America**		
		Canada	
		United States	
			Alabama
			Alaska
			Arizona
			Arkansas

Super regions	Regions	Country names	
			California
			Colorado
			Connecticut
			Delaware
			District of Columbia
			Florida
			Georgia
			Hawaii
			Idaho
			Illinois
			Indiana
			Iowa
			Kansas
			Kentucky
			Louisiana
			Maine
			Maryland
			Massachusetts
			Michigan
			Minnesota
			Mississippi
			Missouri
			Montana
			Nebraska
			Nevada
			New Hampshire
			New Jersey
			New Mexico
			New York
			North Carolina
			North Dakota
			Ohio
			Oklahoma
			Oregon
			Pennsylvania
			Rhode Island
			South Carolina
			South Dakota
			Tennessee
			Texas
			Utah
			Vermont
			Virginia
			Washington
			West Virginia
			Wisconsin
			Wyoming
	**Southern Latin America**		
		Argentina	
		Chile	
		Uruguay	
	**Western Europe**		

Super regions	Regions	Country names	
		Andorra	
		Austria	
		Belgium	
		Cyprus	
		Denmark	
		Finland	
		France	
		Germany	
		Greece	
		Greenland	
		Iceland	
		Ireland	
		Israel	
		Italy	
		Luxembourg	
		Malta	
		Netherlands	
		Norway	
		Portugal	
		Spain	
		Sweden	
		Switzerland	
		United Kingdom	
			England
			Northern Ireland
			Scotland
			Wales
**Latin America and Caribbean**			
	**Andean Latin America**		
		Bolivia	
		Ecuador	
		Peru	
	**Caribbean**		
		Antigua and Barbuda	
		The Bahamas	
		Barbados	
		Belize	
		Bermuda	
		Cuba	
		Dominica	
		Dominican Republic	
		Grenada	
		Guyana	
		Haiti	
		Jamaica	
		Puerto Rico	
		Saint Lucia	
		Saint Vincent and the Grenadines	
		Suriname	
		Trinidad and Tobago	
	**Central Latin America**		
		Colombia	

Super regions	Regions	Country names	
		Costa Rica	
		El Salvador	
		Guatemala	
		Honduras	
		Mexico	
			Aguascalientes
			Baja California
			Baja California Sur
			Campeche
			Coahuila
			Colima
			Chiapas
			Chihuahua
			Distrito Federal
			Durango
			Guanajuato
			Guerrero
			Hidalgo
			Jalisco
			México
			Michoacán de Ocampo
			Morelos
			Nayarit
			Nuevo León
			Oaxaca
			Puebla
			Querétaro
			Quintana Roo
			San Luis Potosí
			Sinaloa
			Sonora
			Tabasco
			Tamaulipas
			Tlaxcala
			Veracruz de Ignacio de la Llave
			Yucatán
			Zacatecas
		Nicaragua	
		Panama	
		Venezuela	
	**Tropical Latin America**		
		Brazil	
			Acre
			Alagoas
			Amapá
			Amazonas
			Bahia
			Ceará
			Distrito Federal
			Espírito Santo
			Goiás
			Maranhão

Super regions	Regions	Country names	
			Mato Grosso
			Mato Grosso do Sul
			Minas Gerais
			Pará
			Paraíba
			Paraná
			Pernambuco
			Piauí
			Rio de Janeiro
			Rio Grande do Norte
			Rio Grande do Sul
			Rondônia
			Roraima
			Santa Catarina
			São Paulo
			Sergipe
			Tocantins
		Paraguay	
**North Africa and Middle East**			
	**North Africa and Middle East**		
		Afghanistan	
		Algeria	
		Bahrain	
		Egypt	
		Iran	
		Iraq	
		Jordan	
		Kuwait	
		Lebanon	
		Libya	
		Morocco	
		Palestine	
		Oman	
		Qatar	
		Saudi Arabia	
			Riyadh
			Makkah
			Al Madinah
			Al Qasim
			Al Sharqia
			Asir
			Tabuk
			Ha'il
			Al Hudud ash Shamaliyah
			Jizan
			Najran
			Al Bahah
			Al Jawf
		Sudan	
		Syria	
		Tunisia	
		Turkey	

Super regions	Regions	Country names	
		United Arab Emirates	
		Yemen	
**South Asia**			
	**South Asia**		
		Bangladesh	
		Bhutan	
		India	
			Andhra Pradesh
			Arunachal Pradesh
			Assam
			Bihar
			Chhattisgarh
			Goa
			Gujarat
			Haryana
			Himachal Pradesh
			Jammu and Kashmir
			Jharkhand
			Karnataka
			Kerala
			Madhya Pradesh
			Maharashtra
			Manipur
			Meghalaya
			Mizoram
			Nagaland
			Orissa
			Punjab
			Rajasthan
			Sikkim
			Tamil Nadu
			Telangana
			Tripura
			Uttar Pradesh
			Uttarakhand
			West Bengal
		Nepal	
		Pakistan	
**Sub-Saharan Africa**			
	**Central Sub-Saharan Africa**		
		Angola	
		Central African Republic	
		Congo	
		Democratic Republic of the Congo	
		Equatorial Guinea	
		Gabon	
	**Eastern Sub-Saharan Africa**		
		Burundi	
		Comoros	
		Djibouti	
		Eritrea	

Super regions	Regions	Country names	
		Ethiopia	
		Kenya	
		Madagascar	
		Malawi	
		Mozambique	
		Rwanda	
		Somalia	
		South Sudan	
		Tanzania	
		Uganda	
		Zambia	
	**Southern Sub-Saharan Africa**		
		Botswana	
		Lesotho	
		Namibia	
		South Africa	
			Eastern Cape
			Free State
			Gauteng
			KwaZulu-Natal
			Limpopo
			Mpumalanga
			North-West
			Northern Cape
			Western Cape
		Swaziland	
		Zimbabwe	
	**Western Sub-Saharan Africa**		
		Benin	
		Burkina Faso	
		Cameroon	
		Cape Verde	
		Chad	
		Cote d'Ivoire	
		The Gambia	
		Ghana	
		Guinea	
		Guinea-Bissau	
		Liberia	
		Mali	
		Mauritania	
		Niger	
		Nigeria	
		Sao Tome and Principe	
		Senegal	
		Sierra Leone	
		Togo	
**Southeast Asia, East Asia, and Oceania**			
	**East Asia**		
		China	
			Anhui

Super regions	Regions	Country names	
			Beijing
			Chongqing
			Fujian
			Gansu
			Guangdong
			Guangxi
			Guizhou
			Hainan
			Hebei
			Heilongjiang
			Henan
			Hong Kong Special Administrative Region of China
			Hubei
			Hunan
			Inner Mongolia
			Jiangsu
			Jiangxi
			Jilin
			Liaoning
			Macao Special Administrative Region of China
			Ningxia
			Qinghai
			Shaanxi
			Shandong
			Shanghai
			Shanxi
			Sichuan
			Tianjin
			Tibet
			Xinjiang
			Yunnan
			Zhejiang
		North Korea	
		Taiwan	
	**Southeast Asia**		
		Cambodia	
		Indonesia	
		Laos	
		Malaysia	
		Maldives	
		Mauritius	
		Myanmar	
		Philippines	
		Seychelles	
		Sri Lanka	
		Thailand	
		Timor-Leste	
		Vietnam	
	**Oceania**		
		American Samoa	
		Federated States of Micronesia	
		Fiji	

Super regions	Regions	Country names
		Guam
		Kiribati
		Marshall Islands
		Papua New Guinea
		Samoa
		Solomon Islands
		Tonga
		Vanuatu
