# Supplementary material for: Cervical cancer burden and attributable risk factors across different age and regions from 1990 to 2021 and future burden prediction: results from the global burden of disease study 2021
Source: Front Oncol. 2025 Feb 7;15:1541452. doi: 10.3389/fonc.2025.1541452 (PMC11842224; doi:10.3389/fonc.2025.1541452)
Supplement: Supplementary file 3 [file Table1.doc]

**Table S1. National incidences and** mortalities of cervical cancer in 2021.

| **Location** | **Incidence** | | **Mortality** | |
| --- | --- | --- | --- | --- |
|  | ***n* (95% UI)** | **Rate (95% UI)** | ***n* (95%UI)** | **Rate (95% UI)** |
| China | 132,787.82 (95,959.18, 172,599.73) | 13.37 (9.61, 17.51) | 49,841.19 (36,878.07, 64,386.31) | 4.64 (3.44, 6.00) |
| North Korea | 3,034.83 (2,072.17, 4,285.80) | 17.45 (11.82, 24.70) | 1,396.91 (982.34, 2,016.48) | 7.60 (5.30, 10.91) |
| Taiwan | 2,041.14 (1,795.91, 2,291.87) | 10.94 (9.57, 12.21) | 793.97 (692.86, 895.71) | 3.67 (3.21, 4.13) |
| Cambodia | 1,389.08 (1,013.08, 1,934.72) | 17.05 (12.51, 23.86) | 733.09 (543.22, 1,022.10) | 9.45 (7.08, 13.17) |
| Indonesia | 20,549.61 (14,948.41, 27,288.43) | 13.78 (10.07, 18.15) | 10,302.39 (7,446.36, 13,508.82) | 7.38 (5.39, 9.53) |
| Laos | 460.00 (327.74, 599.02) | 14.98 (10.77, 19.32) | 243.80 (175.96, 323.86) | 8.71 (6.37, 11.47) |
| Malaysia | 2,994.93 (2,322.06, 3,526.66) | 19.44 (15.08, 22.81) | 1,367.09 (1,057.52, 1,595.83) | 9.45 (7.27, 11.02) |
| Maldives | 11.64 (8.85, 14.60) | 5.98 (4.56, 7.53) | 4.41 (3.34, 5.63) | 2.68 (2.06, 3.45) |
| Myanmar | 3,934.26 (2,799.89, 5,410.71) | 13.07 (9.29, 17.92) | 2,064.04 (1,490.57, 2,918.85) | 7.04 (5.09, 9.97) |
| Philippines | 6,636.04 (4,985.40, 8,521.51) | 12.94 (9.80, 16.55) | 3,147.37 (2,436.43, 3,967.21) | 6.47 (5.05, 8.11) |
| Sri Lanka | 1,142.45 (717.00, 1,608.76) | 7.98 (5.04, 11.14) | 525.06 (341.81, 734.84) | 3.53 (2.30, 4.91) |
| Thailand | 10,962.99 (8,328.79, 14,225.04) | 21.93 (16.54, 28.65) | 4,617.49 (3,484.21, 5,994.67) | 8.25 (6.23, 10.64) |
| Timor-Leste | 64.82 (46.70, 87.86) | 13.73 (9.95, 18.56) | 37.03 (27.70, 50.17) | 8.22 (6.12, 11.11) |
| Vietnam | 9,655.20 (7,258.60, 12,830.44) | 16.50 (12.49, 21.83) | 4,369.00 (3,345.03, 5,713.47) | 7.66 (5.90, 9.94) |
| Fiji | 189.44 (132.07, 255.08) | 42.64 (29.70, 57.21) | 92.55 (64.63, 124.74) | 22.33 (15.73, 29.35) |
| Kiribati | 34.40 (24.39, 47.24) | 70.03 (50.27, 93.83) | 19.58 (14.00, 26.00) | 45.10 (32.22, 58.73) |
| Marshall Islands | 8.20 (5.08, 11.53) | 34.03 (21.47, 46.90) | 3.86 (2.42, 5.29) | 18.28 (12.03, 24.61) |
| Federated States of Micronesia | 13.97 (9.80, 19.73) | 31.05 (22.09, 43.65) | 6.81 (4.86, 9.35) | 16.41 (12.00, 22.27) |
| Papua New Guinea | 880.19 (630.74, 1,499.04) | 24.52 (17.62, 41.84) | 423.32 (298.42, 731.83) | 14.00 (9.87, 23.93) |
| Samoa | 22.17 (14.92, 30.16) | 26.91 (18.14, 36.65) | 9.16 (6.31, 12.29) | 11.75 (8.15, 15.75) |
| Solomon Islands | 79.93 (57.47, 115.59) | 32.69 (23.81, 45.97) | 39.10 (28.22, 55.93) | 18.40 (13.68, 25.39) |
| Tonga | 16.43 (11.76, 22.23) | 36.86 (26.45, 49.69) | 8.14 (5.93, 10.68) | 18.63 (13.54, 24.44) |
| Vanuatu | 28.07 (20.50, 37.56) | 24.39 (17.99, 32.33) | 14.07 (10.50, 18.38) | 13.88 (10.40, 18.16) |
| Armenia | 243.72 (214.07, 279.50) | 11.26 (9.84, 12.93) | 118.65 (105.22, 134.45) | 5.00 (4.43, 5.67) |
| Azerbaijan | 549.30 (413.70, 697.30) | 8.73 (6.60, 11.01) | 247.79 (189.63, 310.53) | 4.11 (3.17, 5.12) |
| Georgia | 454.68 (389.97, 522.43) | 16.88 (14.41, 19.48) | 238.96 (207.04, 275.10) | 7.63 (6.60, 8.76) |
| Kazakhstan | 1,750.56 (1,479.12, 2,025.75) | 16.20 (13.69, 18.77) | 632.69 (539.92, 738.36) | 5.88 (5.02, 6.85) |
| Kyrgyzstan | 595.39 (463.34, 748.17) | 18.64 (14.65, 23.18) | 245.94 (199.70, 300.14) | 8.22 (6.70, 9.92) |
| Mongolia | 297.52 (221.91, 393.53) | 18.35 (13.61, 24.61) | 139.67 (103.64, 184.29) | 9.64 (7.14, 12.68) |
| Tajikistan | 272.20 (182.16, 403.55) | 6.88 (4.70, 9.89) | 130.20 (90.51, 185.95) | 3.78 (2.72, 5.21) |
| Turkmenistan | 413.92 (306.11, 546.63) | 16.53 (12.26, 21.79) | 180.72 (134.19, 241.38) | 7.43 (5.55, 9.90) |
| Uzbekistan | 2,601.54 (2,101.29, 3,108.86) | 14.99 (12.14, 17.92) | 1158.76 (958.21, 1394.14) | 7.11 (5.89, 8.55) |
| Albania | 133.97 (89.33, 194.25) | 7.71 (5.12, 11.39) | 53.52 (36.88, 74.80) | 2.59 (1.76, 3.65) |
| Bosnia and Herzegovina | 337.45 (246.61, 432.07) | 13.51 (9.64, 17.47) | 143.29 (107.70, 183.25) | 4.68 (3.46, 6.02) |
| Bulgaria | 1,196.34 (975.37, 1,409.27) | 23.22 (18.95, 27.63) | 504.26 (415.98, 595.04) | 7.80 (6.43, 9.17) |
| Croatia | 458.26 (353.26, 578.21) | 13.31 (10.21, 16.84) | 157.60 (125.09, 193.96) | 3.51 (2.74, 4.35) |
| Czech Republic | 795.37 (673.57, 939.00) | 9.60 (8.13, 11.29) | 391.86 (325.62, 470.41) | 3.70 (3.11, 4.40) |
| Hungary | 1,161.78 (948.93, 1,425.27) | 15.75 (12.53, 19.59) | 450.21 (379.70, 529.13) | 4.73 (3.94, 5.63) |
| Macedonia | 226.28 (166.51, 297.97) | 14.86 (10.96, 19.46) | 91.86 (67.93, 119.99) | 5.64 (4.20, 7.33) |
| Montenegro | 61.22 (47.88, 76.95) | 14.50 (11.30, 18.41) | 22.25 (17.36, 27.67) | 4.50 (3.51, 5.58) |
| Poland | 3,337.80 (2,956.52, 3,721.28) | 10.63 (9.34, 11.88) | 1,957.70 (1,734.91, 2,161.95) | 5.17 (4.58, 5.72) |
| Romania | 4,023.00 (3,452.68, 4,596.82) | 27.86 (23.77, 32.03) | 1,841.23 (1,619.89, 2,078.73) | 10.45 (9.14, 11.90) |
| Serbia | 1,391.97 (1,083.75, 1,766.62) | 21.29 (16.31, 27.21) | 595.70 (465.92, 756.18) | 7.54 (5.88, 9.56) |
| Slovakia | 583.90 (435.96, 747.08) | 14.47 (10.90, 18.48) | 231.40 (171.99, 301.98) | 4.83 (3.61, 6.24) |
| Slovenia | 288.95 (233.22, 345.81) | 20.02 (15.90, 24.25) | 52.75 (43.42, 62.15) | 2.38 (1.95, 2.80) |
| Belarus | 946.72 (750.07, 1,181.98) | 13.54 (10.58, 17.04) | 388.72 (314.69, 471.86) | 4.66 (3.74, 5.73) |
| Estonia | 165.84 (141.21, 193.22) | 15.43 (12.98, 17.98) | 65.17 (55.69, 75.32) | 4.56 (3.88, 5.28) |
| Latvia | 163.00 (137.06, 187.66) | 9.74 (8.11, 11.42) | 99.56 (84.55, 114.85) | 4.66 (3.99, 5.39) |
| Lithuania | 382.01 (327.52, 441.30) | 16.75 (14.30, 19.55) | 175.71 (150.36, 200.50) | 5.72 (4.87, 6.55) |
| Moldova | 423.82 (372.75, 487.09) | 15.28 (13.36, 17.56) | 202.28 (181.05, 227.17) | 6.49 (5.79, 7.29) |
| Russian Federation | 20,285.83 (17,941.92, 22,495.50) | 19.39 (17.08, 21.57) | 7,490.92 (6,660.36, 8,246.03) | 6.14 (5.44, 6.80) |
| Ukraine | 2,972.03 (1,871.30, 4,373.94) | 7.93 (4.84, 11.97) | 1,506.28 (967.24, 2,146.95) | 3.56 (2.24, 5.19) |
| Brunei | 41.20 (31.93, 52.09) | 17.94 (14.11, 22.45) | 15.99 (12.52, 19.91) | 7.90 (6.32, 9.67) |
| Japan | 11,820.39 (10,737.16, 12,614.19) | 12.35 (11.56, 13.11) | 3,891.34 (3,288.82, 4,253.26) | 2.74 (2.52, 2.88) |
| South Korea | 3,420.89 (2,693.30, 4,462.01) | 8.88 (7.12, 11.47) | 1,074.61 (831.54, 1,386.28) | 2.25 (1.77, 2.89) |
| Singapore | 295.36 (265.88, 326.27) | 7.19 (6.50, 7.95) | 92.14 (82.04, 101.32) | 2.13 (1.90, 2.34) |
| Australia | 1,573.65 (1,406.14, 1,742.32) | 9.11 (8.14, 10.16) | 405.91 (355.94, 449.91) | 1.89 (1.67, 2.09) |
| New Zealand | 177.91 (160.92, 198.52) | 5.40 (4.81, 6.09) | 62.68 (55.72, 68.92) | 1.62 (1.47, 1.78) |
| Andorra | 2.82 (1.84, 4.19) | 4.53 (2.92, 6.73) | 0.94 (0.65, 1.34) | 1.25 (0.86, 1.81) |
| Austria | 475.93 (422.58, 522.57) | 6.75 (6.06, 7.44) | 205.05 (179.03, 226.50) | 2.23 (1.97, 2.44) |
| Belgium | 654.03 (584.74, 727.12) | 7.89 (6.97, 8.87) | 273.07 (235.41, 303.36) | 2.39 (2.14, 2.65) |
| Cyprus | 62.28 (49.49, 76.21) | 6.43 (5.15, 7.89) | 23.76 (18.92, 29.21) | 2.31 (1.84, 2.83) |
| Denmark | 350.30 (314.95, 384.45) | 8.47 (7.61, 9.39) | 168.63 (147.24, 186.58) | 2.88 (2.58, 3.15) |
| Finland | 217.08 (190.96, 243.40) | 5.44 (4.78, 6.10) | 80.74 (68.58, 91.12) | 1.35 (1.19, 1.50) |
| France | 3,955.84 (3,558.98, 4,341.75) | 8.05 (7.28, 9.01) | 1,745.45 (1,489.32, 1,957.44) | 2.42 (2.16, 2.67) |
| Germany | 5,906.92 (5,284.73, 6,444.70) | 9.31 (8.35, 10.31) | 2,301.14 (2,025.83, 2,545.12) | 2.61 (2.37, 2.83) |
| Greece | 695.15 (631.49, 750.23) | 8.28 (7.58, 8.93) | 326.45 (287.79, 356.06) | 2.78 (2.53, 2.99) |
| Iceland | 15.23 (13.26, 17.47) | 6.86 (5.87, 7.99) | 5.26 (4.46, 5.91) | 1.86 (1.61, 2.09) |
| Ireland | 261.20 (229.34, 292.29) | 8.02 (7.04, 8.98) | 82.74 (72.03, 92.76) | 2.15 (1.90, 2.40) |
| Israel | 297.02 (263.07, 331.06) | 5.38 (4.76, 6.03) | 127.42 (109.64, 142.48) | 1.97 (1.73, 2.19) |
| Italy | 4,337.46 (3,915.16, 4,678.59) | 8.38 (7.79, 8.99) | 1,535.74 (1,325.22, 1,681.33) | 2.13 (1.92, 2.29) |
| Luxembourg | 22.97 (20.39, 25.60) | 4.99 (4.43, 5.57) | 9.10 (8.09, 10.13) | 1.64 (1.47, 1.82) |
| Malta | 18.69 (16.23, 21.13) | 5.62 (4.87, 6.40) | 7.92 (6.83, 9.12) | 1.74 (1.52, 1.98) |
| Netherlands | 899.09 (801.01, 1,005.60) | 7.26 (6.42, 8.20) | 311.39 (278.07, 348.61) | 1.85 (1.67, 2.07) |
| Norway | 330.71 (304.38, 355.24) | 9.03 (8.34, 9.72) | 143.87 (125.99, 156.40) | 2.86 (2.60, 3.07) |
| Portugal | 1,025.10 (913.98, 1,129.86) | 11.73 (10.56, 13.02) | 358.78 (313.72, 400.87) | 2.95 (2.64, 3.23) |
| Spain | 3,147.81 (2,806.67, 3,507.13) | 8.67 (7.77, 9.64) | 922.55 (802.11, 1,028.32) | 1.92 (1.72, 2.12) |
| Sweden | 487.88 (416.41, 560.26) | 6.63 (5.64, 7.72) | 264.76 (220.76, 301.38) | 2.38 (2.03, 2.72) |
| Switzerland | 356.29 (314.78, 396.73) | 5.42 (4.85, 6.06) | 141.34 (116.91, 159.73) | 1.52 (1.33, 1.70) |
| United Kingdom | 4,373.29 (4,165.53, 4,563.01) | 10.33 (9.88, 10.81) | 1,315.42 (1,206.56, 1,377.08) | 2.20 (2.07, 2.28) |
| Argentina | 6,606.16 (6,018.47, 7,294.54) | 24.66 (22.43, 27.27) | 2,764.52 (2,511.28, 3,053.04) | 9.59 (8.74, 10.54) |
| Chile | 2,181.08 (1,959.58, 2,419.22) | 18.50 (16.49, 20.60) | 834.78 (762.58, 909.81) | 6.29 (5.76, 6.84) |
| Uruguay | 514.36 (464.57, 571.67) | 23.13 (20.80, 25.81) | 232.72 (208.58, 256.16) | 8.53 (7.72, 9.36) |
| Canada | 3,146.06 (2,837.17, 3,429.57) | 13.23 (11.78, 14.59) | 758.79 (684.48, 830.56) | 2.28 (2.10, 2.47) |
| United States | 27,261.62 (26,016.42, 28,397.05) | 12.64 (12.16, 13.16) | 7,203.16 (6,683.44, 7,552.04) | 2.69 (2.54, 2.80) |
| Antigua and Barbuda | 13.51 (12.44, 14.65) | 23.46 (21.61, 25.43) | 5.73 (5.34, 6.14) | 10.04 (9.39, 10.72) |
| The Bahamas | 63.26 (49.05, 80.01) | 27.04 (20.99, 34.17) | 24.95 (19.63, 31.58) | 10.82 (8.56, 13.66) |
| Barbados | 71.83 (56.31, 91.04) | 31.43 (24.44, 39.88) | 33.52 (26.60, 41.56) | 12.79 (10.16, 15.94) |
| Belize | 74.84 (64.21, 85.74) | 39.66 (34.35, 45.27) | 29.86 (26.17, 34.12) | 17.63 (15.51, 20.00) |
| Cuba | 1,833.60 (1,523.14, 2,162.93) | 22.42 (18.50, 26.55) | 719.76 (605.33, 837.15) | 7.51 (6.32, 8.80) |
| Dominica | 12.53 (9.45, 16.11) | 31.42 (23.66, 40.48) | 6.57 (5.11, 8.33) | 15.50 (12.01, 19.67) |
| Dominican Republic | 1,221.88 (885.34, 1,615.11) | 22.40 (16.19, 29.68) | 547.67 (401.98, 714.10) | 10.30 (7.55, 13.42) |
| Grenada | 22.45 (19.23, 26.59) | 39.17 (33.26, 46.33) | 10.31 (8.97, 11.97) | 17.46 (15.17, 20.27) |
| Guyana | 121.64 (87.52, 166.32) | 32.36 (23.52, 44.10) | 60.85 (45.82, 80.01) | 16.87 (12.78, 21.99) |
| Haiti | 2,406.59 (1,618.00, 3,376.69) | 45.94 (31.09, 64.15) | 1,304.35 (889.31, 1,818.80) | 28.28 (19.39, 39.02) |
| Jamaica | 542.72 (399.18, 723.99) | 34.40 (25.19, 45.93) | 224.81 (169.55, 288.37) | 13.98 (10.53, 17.99) |
| Saint Lucia | 34.31 (27.47, 41.89) | 29.38 (23.48, 35.98) | 15.46 (12.43, 18.54) | 12.50 (10.06, 14.99) |
| Saint Vincent and the Grenadines | 27.64 (23.58, 32.19) | 42.23 (36.11, 49.24) | 12.45 (10.79, 14.40) | 18.36 (15.92, 21.25) |
| Suriname | 105.69 (77.03, 136.05) | 31.93 (23.32, 41.19) | 50.36 (37.15, 63.93) | 14.87 (10.95, 18.85) |
| Trinidad and Tobago | 258.95 (194.01, 338.35) | 28.90 (21.50, 38.01) | 110.96 (84.38, 141.73) | 11.61 (8.86, 14.85) |
| Bolivia | 1,941.80 (1,327.25, 2,753.32) | 36.84 (25.27, 51.58) | 1,093.87 (741.12, 1,524.87) | 22.05 (15.03, 30.41) |
| Ecuador | 2,120.34(1,624.53, 2,695.63) | 23.78 (18.21, 30.20) | 980.80 (762.01, 1,227.69) | 11.25 (8.75, 14.06) |
| Peru | 5,695.02 (4,059.69, 7,555.89) | 30.77 (21.95, 40.85) | 2,360.64 (1,697.93, 3,104.51) | 13.17 (9.51, 17.37) |
| Colombia | 8,151.03 (6,664.88, 9,861.22) | 28.50 (23.20, 34.55) | 2,331.27 (1,934.94, 2,782.88) | 7.87 (6.51, 9.41) |
| Costa Rica | 699.89 (599.00, 803.59) | 24.27 (20.72, 27.93) | 185.38 (161.78, 211.58) | 6.28 (5.48, 7.17) |
| El Salvador | 1,594.96 (1,200.39, 2,044.22) | 45.54 (34.26, 58.40) | 549.16 (425.23, 690.20) | 15.36 (11.91, 19.26) |
| Guatemala | 2,339.29 (1,997.50, 2,747.39) | 34.72(29.76, 40.74) | 920.00 (790.57, 1,073.98) | 14.76 (12.75, 17.17) |
| Honduras | 1,372.10 (817.68, 2,007.81) | 33.62 (20.76, 48.16) | 574.62 (368.13, 801.90) | 15.66 (10.06, 21.50) |
| Mexico | 16,469.24 (13,699.44, 19,269.83) | 22.99 (19.14, 26.86) | 5,471.63 (4,608.20, 6,348.30) | 7.87 (6.64, 9.12) |
| Nicaragua | 1,035.43 (803.33, 1,340.05) | 33.30 (25.96, 42.94) | 339.64 (270.10, 439.62) | 11.89 (9.55, 15.30) |
| Panama | 690.23 (548.76, 841.23) | 31.31 (24.85, 38.21) | 191.62 (151.72, 232.27) | 8.44 (6.68, 10.24) |
| Venezuela | 7,990.82 (5,727.09, 10,528.19) | 50.15 (35.91, 66.02) | 2,544.89 (1,869.50, 3,307.49) | 15.72 (11.54, 20.43) |
| Brazil | 26,626.44 (25,118.07, 27,983.43) | 19.90 (18.79, 20.90) | 11,248.38 (10,507.97, 11,875.54) | 8.21 (7.68, 8.66) |
| Paraguay | 1,196.51 (873.80, 1,638.87) | 35.59 (26.12, 48.68) | 510.54 (379.21, 681.90) | 16.04 (11.94, 21.38) |
| Algeria | 1,578.38 (1,217.49, 2,041.84) | 7.43 (5.78, 9.54) | 683.38 (541.84, 859.84) | 3.69 (2.96, 4.59) |
| Bahrain | 23.39 (17.87, 30.70) | 4.65 (3.59, 6.06) | 9.65 (7.24, 12.70) | 2.44 (1.88, 3.17) |
| Egypt | 1,332.62 (962.69, 1,755.23) | 3.63 (2.69, 4.71) | 696.95 (498.86, 907.45) | 2.29 (1.71, 2.86) |
| Iran | 1,123.10 (969.37, 1,284.77) | 2.50 (2.16, 2.85) | 554.62 (478.07, 628.60) | 1.37 (1.18, 1.55) |
| Iraq | 542.64 (388.33, 751.97) | 3.51 (2.51, 4.80) | 253.91 (181.32, 340.67) | 1.86 (1.33, 2.48) |
| Jordan | 136.52 (98.57, 186.36) | 2.95 (2.15, 3.96) | 57.09 (42.04, 75.80) | 1.47 (1.11, 1.90) |
| Kuwait | 48.64 (40.69, 58.55) | 2.11 (1.78, 2.50) | 14.81 (12.53, 17.52) | 0.92 (0.77, 1.09) |
| Lebanon | 119.88 (91.55, 152.41) | 3.75 (2.86, 4.78) | 53.70 (41.94, 66.40) | 1.66 (1.29, 2.05) |
| Libya | 432.76 (303.41, 597.19) | 12.19 (8.71, 16.81) | 188.96 (135.51, 255.98) | 6.07 (4.43, 8.19) |
| Morocco | 1,985.72 (1,326.88, 2,719.07) | 10.26 (6.93, 13.91) | 1,082.94 (732.90, 1,451.35) | 5.79 (4.00, 7.66) |
| Palestine | 27.42 (21.47, 34.14) | 1.67 (1.32, 2.07) | 13.49 (10.72, 16.53) | 0.97 (0.78, 1.17) |
| Oman | 46.85 (34.31, 62.08) | 3.37 (2.53, 4.27) | 16.88 (12.67, 21.73) | 1.62 (1.23, 2.08) |
| Qatar | 22.82 (16.47, 31.85) | 3.95 (2.94, 5.29) | 7.04 (5.19, 9.94) | 2.04 (1.55, 2.69) |
| Saudi Arabia | 605.30 (430.25, 854.80) | 3.56 (2.60, 4.90) | 103.92 (75.28, 142.58) | 0.89 (0.66, 1.17) |
| Syria | 210.19 (143.27, 292.72) | 2.86 (1.97, 3.95) | 100.85 (69.06, 139.02) | 1.51 (1.05, 2.04) |
| Tunisia | 408.83 (282.84, 557.36) | 5.79 (4.01, 7.87) | 185.31 (126.71, 249.28) | 2.65 (1.82, 3.55) |
| Turkey | 1,839.97 (1,407.71, 2,365.45) | 3.73 (2.86, 4.77) | 923.43 (712.52, 1,185.56) | 1.86 (1.43, 2.37) |
| United Arab Emirates | 179.77 (133.62, 238.82) | 15.46 (11.61, 19.92) | 77.87 (58.94, 102.92) | 11.96 (8.69, 15.99) |
| Yemen | 488.59 (310.04, 726.32) | 4.91 (3.22, 7.18) | 277.00 (182.45, 398.50) | 3.24 (2.23, 4.61) |
| Afghanistan | 1,102.45 (571.73, 1,653.29) | 13.33 (7.09, 20.01) | 645.79 (336.29, 964.89) | 8.96 (4.87, 13.16) |
| Bangladesh | 11,294.43 (7,719.63, 16,347.82) | 14.41 (9.88, 20.68) | 5,634.33 (3,981.78, 7,846.27) | 7.72 (5.43, 10.66) |
| Bhutan | 52.85 (34.25, 75.05) | 15.64 (10.25, 21.75) | 27.88 (18.66, 37.75) | 8.87 (6.00, 12.01) |
| India | 112,102.51 (95,757.45, 129,057.05) | 16.53 (14.14, 18.99) | 60,040.82 (51,584.35, 69,062.11) | 9.24 (7.96, 10.62) |
| Nepal | 2,297.87 (1,536.36, 3,069.51) | 15.96 (10.93, 21.11) | 1,225.96 (844.76, 1,640.70) | 9.18 (6.42, 12.27) |
| Pakistan | 6,734.34 (4,725.45, 9,318.53) | 7.87 (5.61, 10.71) | 3,385.61 (2,417.84, 4,577.84) | 4.66 (3.34, 6.25) |
| Angola | 3,307.61 (2,159.28, 4,885.37) | 35.31 (23.39, 51.56) | 1,825.86 (1,218.52, 2,659.91) | 22.69 (15.19, 32.88) |
| Central African Republic | 755.64 (492.89, 1,087.14) | 44.83 (29.91, 63.82) | 497.84 (327.57, 726.40) | 33.02 (22.16, 47.70) |
| Congo | 946.29 (529.20, 1,422.14) | 47.09 (27.40, 70.25) | 502.35 (290.24, 761.57) | 28.75 (17.08, 42.85) |
| Democratic Republic of the Congo | 9,930.14 (6,533.96, 14,043.36) | 37.92 (25.03, 53.74) | 5,892.24 (3,890.88, 8,345.68) | 25.34 (16.85, 35.65) |
| Equatorial Guinea | 158.94 (86.66, 259.15) | 36.25 (20.29, 57.84) | 73.23 (40.68, 118.00) | 20.39 (12.07, 32.33) |
| Gabon | 229.58 (143.57, 349.12) | 33.24 (21.13, 49.91) | 121.62 (78.21, 179.98) | 19.74 (12.91, 28.90) |
| Burundi | 1,193.84 (821.71, 1,685.55) | 34.89 (24.13, 49.08) | 697.90 (476.92, 990.72) | 23.76 (16.59, 33.30) |
| Comoros | 119.43 (79.22, 167.97) | 38.16 (25.39, 53.63) | 70.22 (46.13, 100.21) | 24.05 (15.80, 34.20) |
| Djibouti | 153.13 (93.13, 235.50) | 34.71 (21.48, 52.64) | 83.27 (51.08, 127.58) | 22.17 (14.03, 33.11) |
| Eritrea | 1,068.38 (667.60, 1,575.24) | 48.85 (30.69, 71.24) | 645.80 (401.04, 931.78) | 32.97 (20.78, 47.02) |
| Ethiopia | 7,884.23 (5,759.64, 11,765.95) | 26.94 (20.07, 38.59) | 4,485.00 (3,356.39, 6,398.21) | 17.92 (13.56, 25.08) |
| Kenya | 3,119.25 (2,194.06, 4,245.06) | 19.35 (13.61, 26.38) | 1,715.66 (1,214.70, 2,347.35) | 12.12 (8.57, 16.38) |
| Madagascar | 3,258.94 (2,002.08, 4,660.86) | 36.33 (22.19, 51.65) | 1,785.99 (1,107.92, 2,479.94) | 22.97 (14.17, 31.64) |
| Malawi | 2,614.42 (1,708.04, 3,698.71) | 44.36 (29.18, 61.73) | 1,376.18 (904.43, 1,924.91) | 27.26 (18.29, 37.60) |
| Mauritius | 117.87 (105.61, 126.49) | 13.38 (11.97, 14.37) | 56.00 (50.35, 59.36) | 5.89 (5.30, 6.25) |
| Mozambique | 4,019.62 (2,441.61, 6,568.68) | 46.72 (28.79, 74.22) | 2,361.28 (1,436.39, 3,807.36) | 31.27 (19.31, 50.23) |
| Rwanda | 1,600.49 (1,030.56, 2,338.10) | 33.79 (22.00, 49.27) | 903.78 (585.08, 1,338.50) | 21.41 (14.07, 31.61) |
| Seychelles | 17.27 (14.31, 20.48) | 29.05 (24.10, 34.39) | 7.93 (6.60, 9.33) | 13.13 (10.87, 15.46) |
| Somalia | 2,581.60 (1,739.58, 3,862.17) | 50.52 (34.42, 73.38) | 1,646.85 (1,122.01, 2,380.25) | 36.56 (25.22, 51.80) |
| Tanzania | 5,989.71 (4,050.20, 8,147.70) | 33.29 (22.99, 44.88) | 3,329.75 (2,293.42, 4,592.72) | 21.11 (14.90, 28.55) |
| Uganda | 4,041.39 (2,748.08, 5,424.43) | 34.78 (24.22, 46.81) | 2,165.22 (1,492.61, 2,933.53) | 21.78 (15.33, 29.15) |
| Zambia | 2,459.26 (1,506.72, 4,627.11) | 45.01 (28.71, 81.92) | 1,288.74 (836.43, 2,349.17) | 27.91 (18.27, 48.36) |
| Botswana | 287.66 (190.71, 473.12) | 28.31 (19.18, 44.69) | 164.08 (113.03, 255.86) | 18.31 (12.81, 28.12) |
| Lesotho | 440.88 (257.63, 643.63) | 60.76 (36.14, 88.56) | 292.33 (176.27, 431.21) | 42.45 (25.97, 62.03) |
| Namibia | 260.44 (162.88, 374.17) | 26.70 (17.11, 37.91) | 139.44 (90.59, 191.61) | 15.86 (10.56, 21.45) |
| South Africa | 11,894.19 (10,380.42, 13,555.19) | 39.99 (34.95, 45.56) | 5,962.43 (5,256.46, 6,760.40) | 21.26 (18.75, 24.11) |
| Swaziland | 219.10 (116.00, 351.37) | 53.27 (28.74, 84.26) | 127.40 (69.02, 200.03) | 34.62 (19.25, 53.61) |
| Zimbabwe | 3,144.41 (2,096.78, 4,446.82) | 59.49 (40.34, 81.99) | 1,838.79 (1,244.73, 2,576.61) | 39.05 (26.94, 53.92) |
| Benin | 935.23 (659.30, 1,232.11) | 25.14 (18.23, 33.01) | 528.40 (385.20, 682.79) | 16.63 (12.21, 21.08) |
| Burkina Faso | 1,868.63 (1,369.79, 2,456.64) | 28.84 (21.39, 37.42) | 1,088.26 (809.03, 1,402.53) | 19.50 (14.65, 25.04) |
| Cameroon | 2,916.32 (1,837.39, 4,142.28) | 31.45 (20.79, 44.42) | 1,547.14 (1,001.40, 2,206.91) | 19.97 (13.34, 28.45) |
| Cape Verde | 54.96 (42.17, 70.47) | 20.71 (15.89, 26.79) | 32.34 (25.07, 40.95) | 12.59 (9.72, 16.05) |
| Chad | 1,321.77 (933.81, 1,765.36) | 34.27 (24.90, 45.34) | 792.72 (576.88, 1,053.99) | 24.23 (18.03, 32.06) |
| Cote d'Ivoire | 1,216.19 (798.91, 1,717.58) | 15.87 (10.54, 22.25) | 656.35 (431.38, 928.82) | 10.20 (6.96, 14.10) |
| The Gambia | 199.83 (128.49, 274.75) | 27.33 (18.70, 36.82) | 100.05 (68.76, 135.41) | 16.22 (11.50, 21.74) |
| Ghana | 3,637.72 (2,486.67, 4,796.43) | 29.20 (19.98, 38.18) | 1,926.35 (1,319.32, 2,527.29) | 17.99 (12.46, 23.59) |
| Guinea | 1,730.52 (1,203.06, 2,369.05) | 43.11 (30.96, 57.99) | 964.34 (689.69, 1,314.19) | 27.90 (20.26, 37.25) |
| Guinea-Bissau | 282.35 (192.65, 370.70) | 46.88 (32.93, 60.31) | 161.25 (112.97, 209.72) | 31.89 (22.42, 40.63) |
| Liberia | 538.39 (357.03, 747.53) | 33.77 (23.34, 46.43) | 274.46 (189.74, 382.72) | 21.18 (14.71, 29.17) |
| Mali | 1,793.42 (1,304.49, 2,410.86) | 28.29 (21.21, 37.38) | 957.99 (722.13, 1,255.33) | 17.81 (13.31, 23.35) |
| Mauritania | 409.00 (288.50, 564.51) | 30.36 (21.35, 41.47) | 219.99 (153.92, 297.27) | 18.53 (13.06, 24.62) |
| Niger | 1,668.34 (1,156.25, 2,396.12) | 29.47 (20.91, 41.45) | 1,018.13 (721.71, 1,440.23) | 20.73 (15.04, 28.90) |
| Nigeria | 12,923.72 (8,036.48, 19,210.00) | 18.50 (11.98, 26.63) | 7,018.04 (4,588.48, 9,989.16) | 11.83 (8.10, 16.36) |
| Sao Tome and Principe | 30.82 (20.90, 42.87) | 40.02 (27.59, 54.17) | 15.33 (10.77, 20.46) | 22.81 (16.04, 30.13) |
| Senegal | 1,540.00 (1,101.84, 2,075.06) | 30.28 (21.81, 40.80) | 877.83 (632.17, 1,186.45) | 19.51 (14.12, 26.28) |
| Sierra Leone | 825.34 (591.58, 1,117.21) | 31.89 (23.18, 42.35) | 458.68 (332.81, 621.97) | 20.84 (15.55, 28.11) |
| Togo | 946.42 (609.64, 1,281.63) | 32.32 (21.20, 43.70) | 519.09 (342.63, 696.23) | 20.57 (14.13, 27.31) |
| American Samoa | 5.52 (4.14, 7.05) | 21.97 (16.44, 27.98) | 2.54 (1.93, 3.25) | 10.51 (7.96, 13.40) |
| Bermuda | 6.01 (4.88, 7.65) | 11.51 (9.29, 14.63) | 2.25 (1.83, 2.85) | 3.26 (2.67, 4.15) |
| Cook Islands | 1.02 (0.76, 1.38) | 8.94 (6.61, 12.05) | 0.42 (0.32, 0.55) | 3.35 (2.50, 4.36) |
| Greenland | 7.07 (5.23, 9.49) | 22.77 (16.59, 30.60) | 2.46 (1.92, 3.27) | 7.59 (5.95, 9.94) |
| Guam | 11.73 (9.85, 13.51) | 12.48 (10.45, 14.39) | 4.56 (3.74, 5.30) | 4.43 (3.66, 5.12) |
| Monaco | 3.06 (2.21, 4.08) | 10.93 (7.63, 15.05) | 1.29 (0.95, 1.71) | 2.89 (2.10, 3.83) |
| Nauru | 1.78 (1.08, 2.54) | 42.10 (26.19, 58.95) | 0.79 (0.49, 1.10) | 21.26 (13.73, 28.90) |
| Niue | 0.24 (0.18, 0.30) | 24.23 (18.65, 30.41) | 0.12 (0.09, 0.15) | 10.52 (8.08, 13.29) |
| Northern Mariana Islands | 9.71 (7.56, 11.77) | 35.36 (28.14, 42.42) | 3.85 (3.06, 4.56) | 15.03 (12.08, 17.40) |
| Palau | 5.85 (4.47, 7.61) | 57.48 (44.17, 74.16) | 2.85 (2.25, 3.63) | 30.03 (23.64, 37.61) |
| Puerto Rico | 296.09 (237.65, 355.54) | 11.83 (9.37, 14.35) | 113.84 (93.25, 137.45) | 3.44 (2.81, 4.14) |
| Saint Kitts and Nevis | 10.05 (8.18, 12.50) | 26.51 (21.75, 32.82) | 4.82 (3.98, 5.85) | 13.12 (10.93, 15.82) |
| San Marino | 0.79 (0.47, 1.24) | 3.09 (1.74, 4.97) | 0.32 (0.20, 0.48) | 0.89 (0.55, 1.39) |
| Tokelau | 0.21 (0.15, 0.26) | 29.57 (22.19, 37.83) | 0.09 (0.07, 0.12) | 12.60 (9.50, 16.22) |
| Tuvalu | 1.68 (1.24, 2.23) | 30.42 (22.49, 40.35) | 0.85 (0.63, 1.11) | 15.62 (11.85, 20.40) |
| Virgin Islands, U.S. | 8.33 (5.86, 11.67) | 13.26 (9.12, 18.93) | 3.83 (2.77, 5.24) | 4.77 (3.44, 6.52) |
| South Sudan | 1,230.34 (825.91, 1,799.06) | 43.28 (29.08, 62.75) | 693.86 (465.80, 1,010.21) | 28.14 (19.33, 40.75) |
| Sudan | 645.03 (375.00, 983.16) | 4.59 (2.85, 6.78) | 314.29 (192.51, 455.56) | 2.71 (1.81, 3.78) |

**Notes:** 95% UI, 95% uncertainty interval

**Table S2. Global and regional mortalities of cervical cancer in 1990 and 2021.**

| **Location** | **1990** | | **2021** | | **EAPC of ASDR (95% CI)** |
| --- | --- | --- | --- | --- | --- |
|  | ***n* (95% UI)** | **ASDR (95% UI)** | ***n* (95% UI)** | **ASDR (95% UI)** |  |
| Global | 211,483.79 (195,724.27, 229,841.01) | 9.68 (8.97, 10.51) | 296,667.24 (272,058.62, 32,1905.72) | 6.62 (6.07, 7.18) | -1.27 (-1.36, -1.18) |
| East Asia | 33,633.49 (27,758.00, 41,119.78) | 7.10 (5.86, 8.64) | 52,032.08 (39,399.83, 66,509.90) | 4.68 (3.55, 5.98) | -1.12 (-1.26, -0.97) |
| Southeast Asia | 16,463.61 (14,232.48, 18,813.30) | 10.70 (9.27, 12.18) | 27,513.07 (23,668.86, 31,860.21) | 7.45 (6.43, 8.59) | -1.32 (-1.43, -1.22) |
| Oceania | 332.56 (260.84, 476.78) | 19.77 (15.75, 27.86) | 662.40 (519.56, 981.79) | 15.27 (12.10, 22.32) | -0.82 (-0.88, -0.77) |
| Central Asia | 2,648.70 (2,529.45, 2,782.16) | 9.53 (9.08, 10.02) | 3,093.38 (2,734.71, 3,497.22) | 6.33 (5.62, 7.13) | -1.06 (-1.21, -0.91) |
| Central Europe | 8,149.02 (7,803.96, 8,455.30) | 10.15 (9.73, 10.54) | 6,589.55 (6,054.53, 7,118.26) | 6.02 (5.53, 6.53) | -1.85 (-2.01, -1.69) |
| Eastern Europe | 12,904.99 (12,416.82, 13,337.74) | 7.58 (7.30, 7.84) | 9,928.64 (8,989.70, 10,954.50) | 5.50 (4.96, 6.07) | -1.22 (-1.31, -1.13) |
| High-income Asia Pacific | 4,740.79 (4,402.87, 5,062.90) | 4.27 (3.96, 4.57) | 5,074.08 (4,303.28, 5,602.42) | 2.53 (2.28, 2.73) | -1.67 (-1.78, -1.57) |
| Australasia | 654.11 (606.91, 712.90) | 5.34 (4.98, 5.83) | 468.60 (413.37, 514.71) | 1.85 (1.66, 2.02) | -3.21 (-3.44, -2.97) |
| Western Europe | 14,370.49 (13,559.11, 14,904.32) | 4.70 (4.48, 4.86) | 10,362.27 (9,191.61, 11,142.66) | 2.29 (2.11, 2.43) | -2.02 (-2.24, -1.80) |
| Southern Latin America | 3,020.03 (2,843.25, 3,200.77) | 12.10 (11.39, 12.83) | 3,832.24 (3,515.12, 4,167.34) | 8.56 (7.89, 9.28) | -1.10 (-1.24, -0.96) |
| High-income North America | 6,834.00 (6,485.64, 7,046.69) | 3.75 (3.59, 3.85) | 7,964.54 (7,391.90, 8,347.93) | 2.64 (2.50, 2.75) | -1.00 (-1.11, -0.89) |
| Caribbean | 2 299.33 (2,027.26, 2,620.95) | 16.36 (14.47, 18.57) | 3,397.30 (2,861.73, 4,026.05) | 12.31 (10.33, 14.68) | -0.88 (-0.96, -0.80) |
| Andean Latin America | 2,390.21 (2,098.72, 2,691.69) | 20.85 (18.31, 23.48) | 4,435.30 (3,432.36, 5,549.87) | 14.02 (10.85, 17.51) | -1.57 (-1.73, -1.42) |
| Central Latin America | 9,518.44 (9,233.33, 9,735.28) | 20.38 (19.63, 20.92) | 13,108.21 (11,285.97, 14,974.14) | 9.52 (8.21, 10.86) | -2.78 (-2.93, -2.64) |
| Tropical Latin America | 6,933.49 (6,633.89, 7,214.09) | 13.25 (12.61, 13.80) | 11,758.92 (10,950.54, 12,428.75) | 8.38 (7.82, 8.86) | -1.78 (-1.90, -1.67) |
| North Africa and Middle East | 3,722.01 (3,235.44, 4,512.26) | 3.96 (3.43, 4.86) | 6,267.72 (5,329.14, 7,273.43) | 2.55 (2.17, 2.93) | -1.39 (-1.44, -1.34) |
| South Asia | 52,119.46 (42,807.81, 60,826.07) | 15.97 (13.02, 18.72) | 70,314.60 (61,026.36, 79,858.14) | 8.72 (7.57, 9.88) | -2.06 (-2.37, -1.75) |
| Central Sub-Saharan Africa | 4,058.65 (3,046.39, 5,271.71) | 28.67 (21.87, 37.26) | 8,913.15 (6,174.97, 12,102.35) | 25.10 (17.45, 33.97) | -0.47 (-0.52, -0.41) |
| Eastern Sub-Saharan Africa | 14,863.70 (12,354.91, 18,151.35) | 32.97 (27.43, 40.13) | 23,269.77 (18,793.24, 29,150.10) | 21.68 (17.79, 26.85) | -1.67 (-1.79, -1.55) |
| Southern Sub-Saharan Africa | 2,940.72 (2,470.56, 3,723.49) | 17.43 (14.74, 22.24) | 8,524.46 (7,477.35, 9,539.89) | 23.90 (21.02, 26.67) | 1.71 (1.22, 2.19) |
| Western Sub-Saharan Africa | 8,885.99 (7,356.84, 10,716.93) | 18.33 (15.26, 21.84) | 19,156.98 (15,041.53, 23,006.99) | 15.57 (12.55, 18.53) | -0.48 (-0.54, -0.41) |
| High-middle SDI | 37,772.38 (35,119.49, 40,433.43) | 6.83 (6.35, 7.31) | 46,300.96 (40,583.48, 52,536.57) | 4.59 (4.02, 5.20) | -1.20 (-1.25, -1.15) |
| High SDI | 29,775.05 (28,337.44, 30,629.55) | 5.03 (4.82, 5.16) | 25,892.52 (23,323.19, 27,329.56) | 2.62 (2.44, 2.74) | -2.05 (-2.16, -1.93) |
| Low-middle SDI | 51,185.71 (44,118.82, 59,057.58) | 14.70 (12.60, 16.94) | 78,178.29 (69,939.81, 86,259.42) | 9.71 (8.67, 10.70) | -1.35 (-1.49, -1.21) |
| Low SDI | 32,109.30 (27,375.10, 39,058.49) | 24.51 (20.89, 29.82) | 49,686.73 (42,207.83, 59,245.87) | 16.36 (13.94, 19.38) | -1.49 (-1.59, -1.38) |
| Middle SDI | 60,363.09 (55,533.71, 65,573.27) | 10.47 (9.64, 11.37) | 96,293.95 (86,730.36, 106,487.33) | 6.72 (6.05, 7.43) | -1.50 (-1.57, -1.44) |

**Notes:** 95% CI, 95% confidence interval; 95% UI, 95% uncertainty interval; ASIR, age-standardized incidence rate; EAPC, estimated annual percentage change; SDI, social-demographic index

**Table S3. Global and regional disability-adjusted life-years for cervical cancer in 1990 and 2021.**

| **Location** | **1990** | | **2021** | | **EAPC of age-standardized** |
| --- | --- | --- | --- | --- | --- |
|  | ***n* (95% UI)** | **Age-standardized DALY rate (95% UI)** | ***n* (95% UI)** | **Age-standardized DALY rate (95% UI)** | **DALY rate (95% CI)** |
| Global | 7,416,286.99 (6,841,378.34, 8,071,399.68) | 330.11 (304.67, 359.10) | 9,911,653.09 (9,053,316.79, 10,798,305.83) | 226.28 (206.51, 246.86) | -1.27 (-1.36, -1.17) |
| East Asia | 1,178,714.01 (958,919.39, 1,452,779.21) | 231.92 (189.37, 285.51) | 1,616,240.23 (1,195,414.47, 2,080,056.88) | 151.15 (111.80, 195.41) | -1.13 (-1.27, -0.98) |
| Southeast Asia | 613,110.37 (526,601.48, 699,789.93) | 362.63 (313.26, 413.29) | 931,500.99 (800,256.95, 1,086,328.05) | 241.92 (207.75, 281.44) | -1.48 (-1.59, -1.37) |
| Oceania | 12,843.92 (9,709.34, 18,346.09) | 654.65 (511.97, 937.93) | 25,245.19 (19,485.62, 37,774.11) | 499.47 (390.49, 739.25) | -0.86 (-0.92, -0.81) |
| Central Asia | 89,741.98 (86,415.34, 94,113.30) | 318.71 (306.47, 334.38) | 108,285.23 (94,717.70, 122,647.14) | 213.84 (187.54, 241.95) | -1.05 (-1.21, -0.89) |
| Central Europe | 263,434.11 (252,754.86, 272,814.86) | 344.13 (330.25, 356.19) | 181,663.84 (166,208.45, 196,949.96) | 191.83 (175.35, 209.14) | -2.05 (-2.24, -1.86) |
| Eastern Europe | 378,040.55 (365,241.83, 390,080.17) | 239.05 (230.85, 246.89) | 320,235.63 (289,145.75, 353,814.91) | 200.77 (180.38, 221.47) | -0.70 (-0.81, -0.59) |
| High-income Asia Pacific | 144,581.72 (135,082.87, 155,151.29) | 133.71 (124.78, 143.46) | 132,808.21 (119,523.11, 144,704.00) | 87.68 (81.63, 94.36) | -1.26 (-1.36, -1.17) |
| Australasia | 20,693.09 (19,367.50, 22,507.02) | 178.43 (166.82, 193.54) | 13,292.50 (12,052.56, 14,533.62) | 61.68 (56.34, 67.50) | -3.13 (-3.40, -2.85) |
| Western Europe | 391,715.41 (376,290.98, 403,410.15) | 148.36 (143.69, 152.94) | 257,407.52 (240,181.20, 272,439.80) | 72.71 (68.94, 76.61) | -2.02 (-2.23, -1.81) |
| Southern Latin America | 103,034.14 (97,005.53, 109,279.28) | 417.99 (393.15, 442.82) | 123,079.06 (114,520.22, 133,155.48) | 296.76 (276.24, 321.05) | -1.08 (-1.22, -0.94) |
| High-income North America | 223,497.32 (215,889.21, 230,643.39) | 133.17 (129.02, 137.29) | 240,100.43 (229,389.99, 250,583.93) | 92.68 (88.88, 96.91) | -1.05 (-1.18, -0.93) |
| Caribbean | 82,426.33 (72,178.87, 94,673.68) | 561.02 (491.47, 642.93) | 115,954.18 (95,882.08, 140,763.70) | 433.17 (356.80, 526.53) | -0.80 (-0.88, -0.72) |
| Andean Latin America | 83,460.23 (73,057.32, 94,115.06) | 667.44 (585.28, 752.00) | 140,300.64 (107,732.87, 176,560.80) | 431.74 (331.78, 543.15) | -1.71 (-1.87, -1.55) |
| Central Latin America | 330,665.11 (322,525.29, 338,290.18) | 627.39 (610.20, 641.61) | 441,929.81 (380,716.90, 508,808.27) | 315.97 (272.32, 363.77) | -2.55 (-2.71, -2.38) |
| Tropical Latin America | 242,801.90 (233,831.96, 252,336.86) | 422.16 (406.02, 438.79) | 395,391.78 (372,666.68, 414,906.51) | 285.57 (269.19, 299.68) | -1.64 (-1.78, -1.49) |
| North Africa and Middle East | 137,468.10 (119,841.29, 167,282.16) | 131.01 (113.82, 159.07) | 217,737.46 (182,978.94, 258,585.89) | 80.04 (67.65, 93.68) | -1.59 (-1.63, -1.56) |
| South Asia | 1,967,172.06 (1,640,297.67, 2,286,747.31) | 552.66 (457.21, 642.70) | 2,432,061.02 (2,097,849.72, 2,773,134.49) | 285.98 (247.23, 325.23) | -2.23 (-2.54, -1.93) |
| Central Sub-Saharan Africa | 150,918.62 (112,943.16, 196,163.40) | 950.65 (718.75, 1233.27) | 330,691.37 (228,131.15, 448,103.50) | 813.59 (562.84, 1,104.29) | -0.54 (-0.59, -0.49) |
| Eastern Sub-Saharan Africa | 564,509.36 (469,048.02, 691,067.32) | 1,117.94 (929.76, 1,367.77) | 876,848.93 (701,727.56, 1,106,311.69) | 709.49 (572.26, 890.60) | -1.81 (-1.93, -1.68) |
| Southern Sub-Saharan Africa | 110,031.68 (92,065.44, 137,135.53) | 600.46 (503.72, 750.80) | 300,881.27 (260,869.60, 338,892.51) | 788.82 (685.47, 885.31) | 1.71 (1.17, 2.26) |
| Western Sub-Saharan Africa | 327,426.99 (269,225.37, 399,723.90) | 608.91 (502.55, 737.46) | 709,997.80 (548,746.35, 865,455.74) | 490.75 (383.61, 591.44) | -0.68 (-0.74, -0.61) |
| High-middle SDI | 1,221,303.54 (1,133,350.07, 1,316,829.43) | 222.34 (206.21, 239.84) | 1,432,107.96 (1,252,617.92, 1,634,548.71) | 152.90 (133.88, 174.32) | -1.12 (-1.17, -1.07) |
| High SDI | 888,170.03 (860,266.04, 911,520.66) | 163.46 (158.83, 167.80) | 707,650.21 (664,622.92, 741,914.34) | 86.41 (82.45, 90.30) | -1.99 (-2.10, -1.88) |
| Low-middle SDI | 1,924,362.37 (1,669,283.01, 2,212,531.72) | 505.86 (438.95, 583.11) | 2,751,152.05 (2,455,069.76, 3,056,560.45) | 321.36 (287.11, 356.77) | -1.48 (-1.62, -1.33) |
| Low SDI | 1,207,229.81 (1,031,665.17, 1,473,493.47) | 833.33 (711.27, 1,016.61) | 1,843,208.46 (1,552,008.40, 2,199,867.45) | 535.11 (454.02, 638.34) | -1.64 (-1.75, -1.53) |
| Middle SDI | 2,165,689.69 (1,982,381.45, 2,359,900.06) | 342.74 (314.41, 372.39) | 3,167,321.58 (2,856,722.06, 3,510,378.41) | 218.95 (197.60, 242.31) | -1.52 (-1.60, -1.44) |

**Notes:** 95% CI, 95% confidence interval; 95% UI, 95% uncertainty interval; DALY, disability-adjusted life year; EAPC, estimated annual percentage change; SDI, social-demographic index.
